# Connexin 43 is required for the maintenance of mitochondrial integrity in brown adipose tissue

**DOI:** 10.1038/s41598-017-07658-y

**Published:** 2017-08-02

**Authors:** Sang-Nam Kim, Hyun-Jung Kwon, Seo-Woo Im, Yeon-Ho Son, Seun Akindehin, Young-Suk Jung, Se Jeong Lee, Im Joo Rhyu, Il Yong Kim, Je-Kyoung Seong, Jinu Lee, Hee-Chan Yoo, James G. Granneman, Yun-Hee Lee

**Affiliations:** 1 College of Pharmacy, Yonsei University, Incheon, 21983 South Korea; 2 College of Pharmacy, Pusan National University, Busan, 46241 South Korea; 3 Department of Anatomy, Korea University College of Medicine, Seoul, 02841 South Korea; 4 Laboratory of Developmental Biology and Genomics, College of Veterinary Medicine, Seoul National University, Korea Mouse Phenotyping Center (KMPC), Seoul, 08826 South Korea; 5 Center for Integrative Metabolic and Endocrine Research, Wayne State University School of Medicine, Detroit, MI USA 48201

## Abstract

We investigated the role of connexin 43 (Cx43) in maintaining the integrity of mitochondria in brown adipose tissue (BAT). The functional effects of Cx43 were evaluated using inducible, adipocyte-specific Cx43 knockout in mice (*Gja1* ^*adipoq*^KO) and by overexpression and knockdown of Cx43 in cultured adipocytes. Mitochondrial morphology was evaluated by electron microscopy and mitochondrial function and autophagy were assessed by immunoblotting, immunohistochemistry, and qPCR. The metabolic effects of adipocyte-specific knockout of Cx43 were assessed during cold stress and following high fat diet feeding. Cx43 expression was higher in BAT compared to white adipose tissue. Treatment with the β3-adrenergic receptor agonist CL316,243 increased Cx43 expression and mitochondrial localization. *Gja1* ^*adipoq*^KO mice reduced mitochondrial density and increased the presence of damaged mitochondria in BAT. Moreover, metabolic activation with CL316,243 further reduced mitochondrial integrity and upregulated autophagy in the BAT of *Gja1* ^*adipoq*^KO mice. Inhibition of Cx43 in cultured adipocytes increased the generation of reactive oxygen species and induction of autophagy during β-adrenergic stimulation. *Gja1* ^*adipoq*^KO mice were cold intolerant, expended less energy in response to β3-adrenergic receptor activation, and were more insulin resistant after a high-fat diet challenge. Collectively, our data demonstrate that Cx43 is required for maintaining the mitochondrial integrity and metabolic activity of BAT.

## Introduction

Brown adipose tissue (BAT) is a specialized thermoregulatory organ whose thermogenic activity is mainly controlled by the sympathetic nervous system^1^. With recent re-discovery of BAT in human and non-canonical pathways to activate thermogenic metabolism, BAT has received renewed interest as a therapeutic target to improve energy balance and metabolic homeostasis^2^; however, mechanisms for efficient activation and maintenance of high levels of oxidation in BAT are not fully understood.

Connexin 43 (Cx43) is a major gap junctional protein that is expressed in multiple tissues^3–5^, and mediates intercellular communication by allowing the passage of small molecules^5^. Brown adipocytes have numerous gap junctions whose numbers increase during cold adaptation^3, 6, 7^. BAT expresses high levels of Cx43 and recent work indicated that cold induced upregulation of Cx43 facilitates neural activation by promoting cyclic AMP coupling among brown adipocytes within inguinal subcutaneous adipose tissue^7^.

In addition to roles of Cx43 in plasma membrane in cell-to cell communication, Cx43 has also been reported to be targeted to subcellular domains^5^. In the heart, Cx43 located in mitochondria is thought to play an important role in protection from ischemia/reperfusion injury of heart, perhaps by regulating mitochondrial ion fluxes and oxidative metabolism^8–10^. Given the similarities in oxidative metabolism in heart and BAT^11^, we examined whether BAT Cx43 might have intracellular function apart from its established role in intercellular communication.

Here, we demonstrated that Cx43 is targeted to mitochondria in adipocytes and that direct pharmacological activation of β3 adrenergic receptors, which appears to bypass the need for intercellular communication, strongly increases expression and mitochondrial targeting of Cx43. We then used adipocyte-specific knockout of Cx43 in adult mice to characterize metabolic phenotype caused by genetic deletion of Cx43. We also studied effects of *in vitro* knockdown of Cx43 in adipocytes to characterize cellular responses and mechanisms that lead to mitochondrial dysfunction upon Cx43 downregulation.

## Results

### Cx43 is the most abundant gap junctional protein in brown adipose tissue

**Figure 1:**
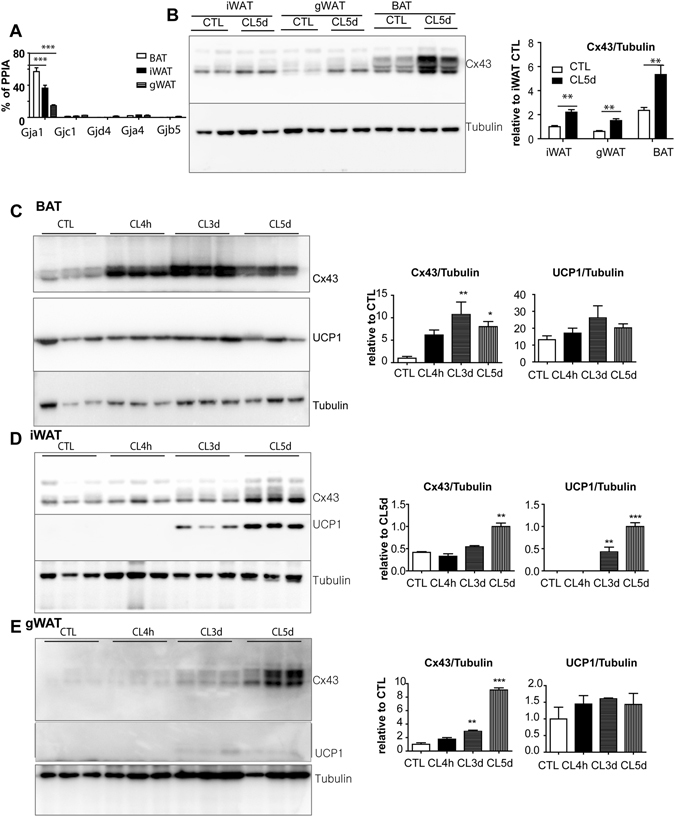
β3-adrenergic stimulation induces Cx43 expression in brown and white adipose tissue. (**A**) qPCR analysis of connexin expression levels in BAT (brown adipose tissue), iWAT (inguinal white adipose tissue), and gWAT (gonadal white adipose tissue) (mean ± S.E.M.; n = 3 per condition, ***P < 0.001). (**B–E**) Immunoblot analysis and quantification of Cx43 and UCP1 expression in BAT, iWAT and gWAT of mice treated with CL up to 5 days. Tubulin was used as a loading control. (mean ± S.E.M.; n = 4 per condition, *p < 0.05, **p < 0.01, ***p < 0.001) (Immunoblots accompanied by size markers in Fig. S6).

Our previous global mRNA profiling of inguinal adipocytes identified a significant expression of several connexins, including *Gja1*, *Gjc1*, *Gjd4*, *Gja4*, and *Gjb5* ^12^. To compare the expression levels of connexin subtypes in white adipose tissue (WAT) and BAT, we performed a quantitative polymerase chain reaction (qPCR) analysis of the connexins in interscapular BAT (iBAT), inguinal WAT (iWAT), and perigonadal WAT (gWAT) (Fig. 1A). In general, *Gja1* (encoding Cx43) was the most abundantly expressed connexin subtype in BAT and WAT (Fig. 1A). BAT had the highest *Gja1* expression, while gWAT had the lowest. Similarly, Cx43 protein was more abundant in BAT than in gWAT and iWAT. Five days of treatment with a selective β3-adrenergic receptor agonist, CL316,243 (CL), significantly increased Cx43 expression in iWAT, gWAT, and BAT (Fig. 1B).

We also examined the time course of the induction of Cx43 and UCP1 proteins by CL treatment (Fig. 1C–E). Five days of CL treatment upregulated Cx43 in BAT, iWAT and gWAT. Simultaneously, an upregulation of UCP1 expression was observed in iWAT, suggesting that Cx43 may be involved in mediating the metabolic activation and browning of WAT induced by β3-adrenergic stimulation. As expected, gWAT did not show a significant upregulation of UCP1 after 5 days of CL treatment, yet the upregulation of Cx43 was significant. The appearance of multiple bands on western blots suggested phosphorylation of Cx43^13^. Collectively, the activation of BAT and browning of WAT induced by β3-adrenergic stimulation were accompanied by increased Cx43 expression, suggesting a potential role of Cx43 in metabolic activation in brown/beige adipocytes.

### β3-adrenergic stimulation increases the mitochondrial localization of Cx43 in adipocytes

**Figure 2:**
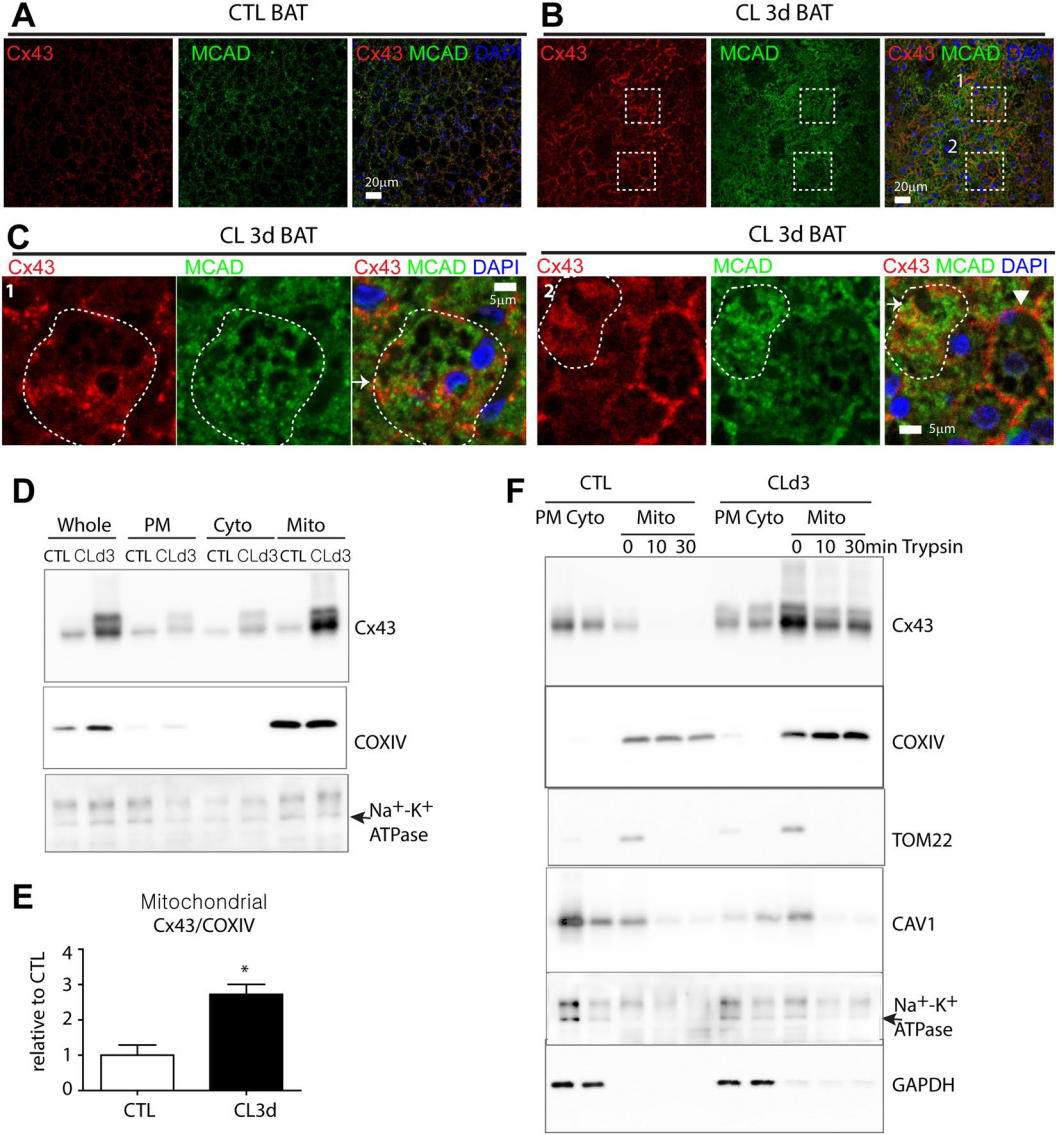
CL316,243 treatment increases mitochondrial localization of Cx43 in brown adipocytes. (**A–C**) Immunohistochemistry of Cx43 and MCAD in paraffin sections of BAT of mice treated with CL for 3 days and untreated controls. Nuclei were counterstained with DAPI. Bars = 5 or 20 μm as indicated. Magnified images of boxed regions of (**B**) are shown in panel (**C**). Arrows indicate close association between Cx43 and MCAD, whereas arrowhead indicates Cx43 expression detected mainly in plasma membrane. (**D,E**) Immunoblot analysis and quantification of Cx43 expression in mitochondrial, plasma membrane and cytosolic fractions of BAT from mice treated with CL for 3 days and untreated controls. (Mean ± S.E.M. n = 3 per condition, *p < 0.05) (**F**) Immunoblot analysis of Cx43 expression in mitochondrial fractions treated with 0.05% trypsin. (Immunoblots accompanied by size markers in Fig. S7).

We examined the distribution of Cx43 expression in BAT by immunofluorescence histochemistry (Fig. 2A and B). As expected Cx43 was detected on brown adipocyte plasma membrane in the BAT of control mice and its expression was increased by treatment with CL for 3 days. After CL treatment, Cx43 was detected in cytosolic location (Fig. 2C). Although distribution of Cx43 is heterogeneous, double label staining for Cx43 and mitochondrial enzyme, medium chain acyl dehydrogenase (MCAD), indicated that Cx43 was also closely associated with some of mitochondria after 3 days of CL treatment (Fig. 2C). To further determine the subcellular localization of Cx43, we performed a western blot analysis of BAT homogenates that were fractionated by differential density centrifugation. CL-induced elevation of Cx43 was largely restricted to heavy membrane fractions containing mitochondria and plasma membrane (Figs 2D and S1). Normalization to a mitochondrial protein, COXIV, indicated significant increase in mitochondrial Cx43 in BAT of CL-treated mice compared to control conditions (Fig. 2E). To enrich for proteins that are targeted to mitochondrial inner membrane, we incubated crude mitochondrial fractions with trypsin, and evaluated protein content of treated fractions following recovery by centrifugation. As shown in Fig. 2F, trypsin incubation depleted the outer mitochondrial membrane protein (i.e. Tom22) as well as plasma membrane markers (e.g. Na^+^/K^+^ -ATPase, Caveolin1). In contrast, Cx43 within the mitochondrial/heavy membrane fraction from BAT of CL-treated mice was partially resistant to trypsin digestion, whereas that from controls was nearly completely removed. Collectively, data suggest that CL treatment increased mitochondrial targeting of Cx43 in brown adipose tissue.

### Adipose-specific *Gja1* knockout reduces mitochondrial density and increases the appearance of damaged mitochondria in BAT

**Figure 3:**
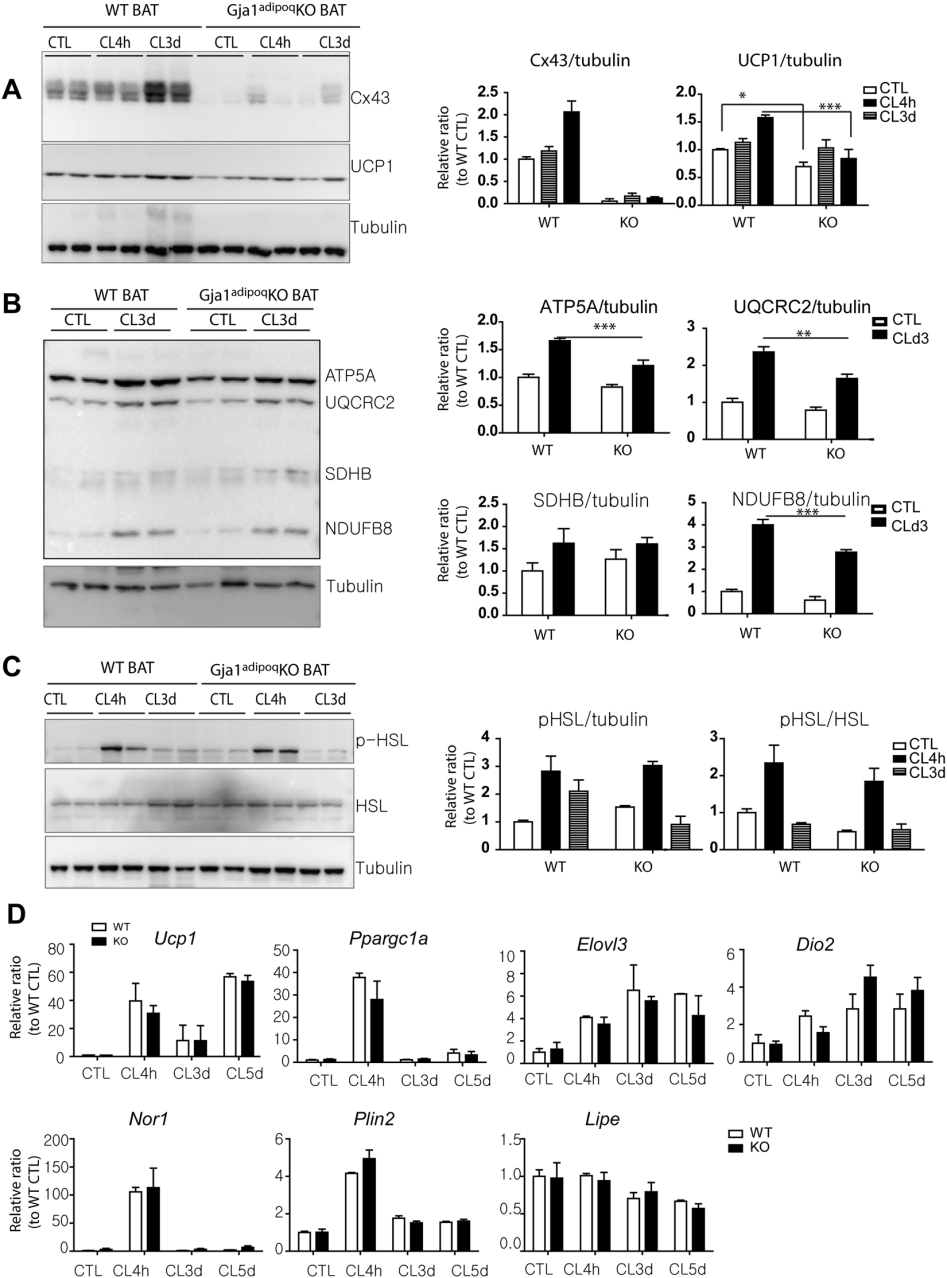
Adipocyte-specific *Gja1* KO reduces mitochondrial contents, but not PKA-downstream signaling in BAT. (**A,B**). Immunoblot analysis of Cx43 and UCP1 expression (**A**) and mitochondrial proteins involved in oxidative phosphorylation (**B**) in BAT of *Gja1* ^*adipoq*^KO mice and WT controls treated with CL up to 3 days. Two-way ANOVA revealed significant main effects of *Gja1* expression in mitochondrial proteins (UCP1: p = 0.0001, ATP5A: p = 0.0004, UQCRC2: p = 0.015, NDUFB8: p = 0. 0004) and significant interaction of genotype and treatment (UCP1: p = 0.0166, NDUFB8: p = 0.0264). Significant differences between WT and KO were determined by post-hoc pairwise comparison with Bonferroni correction (mean ± SEM; n = 4 per condition, *p < 0.01, **p < 0.05, ***p < 0.001). (**C**) Immunoblot analysis of p-HSL and HSL expression in BAT of *Gja1* ^*adipoq*^KO mice and WT controls treated with CL up to 3 days (mean ± SEM; n = 4 per condition). (**D**) qPCR analysis of brown adipocyte markers and genes involved in downstream signaling of PKA activation in BAT of mice treated with CL for up to 5 days and untreated control mice. Two-way ANOVA revealed no significant main effects of *Gja1* expression in mRNA levels (*Ucp1*: p = 0.4829, *Ppargc1a*: p = 0.178, *Elovl3*: p = 0.261, *Dio2*: p = 0.291, *Nor1*: p = 0.583, *Plin2*: p = 0.278, *Lipe*: p = 0.830) (mean ± SEM; n = 4 per condition). (Oil Vehicle controls in Fig. S2, Immunoblots accompanied by size markers in Fig. S8).

To determine the *in vivo* functions of Cx43 in adipose tissue, we inactivated Gja1 in adult mice using adiponectin/CreER-driven recombination. We selected this drug-inducible conditional knockout system to avoid possible chronic or developmental effects. Tamoxifen treatment of Adipoq-CreER/*Gja1* ^flox/flox^ mice effectively deleted CX43 expression, but was without effect in tamoxifen injected mice lacking Cre. Inducible adipocyte-specific *Gja1* ^*adipoq*^KO mice (*Gja1* ^*adipoq*^KO) showed reduced UCP1 protein levels in BAT under basal conditions and after CL stimulation (Fig. 3A). In addition, western blot analysis of mitochondrial proteins indicated a reduction in BAT mitochondrial respiratory chain proteins (Fig. 3A and B). As intended, knockout of Cx43 did not alter the levels of phosphorylation of HSL after 4 h of acute CL treatment, nor did knockout affect the apparent desensitization of adrenergic signalling during chronic treatment (Fig. 3C). In contrast to the reduced mitochondrial UCP1, knockout of Cx43 did not affect induction of UCP1 mRNA levels (Fig. 3D). Similarly, induction of brown adipocyte markers such as *Elovl3* and *Dio2* was not affected by the genetic deletion of *Gja1* (Fig. 3D). In addition, gene downstream of PKA activation (*Nor1* and *Plin2*) did not show any difference in expression between *Gja1* ^*adipoq*^KO and WT mice (Fig. 3D). Thus, the changes in the abundance of mitochondrial proteins, but not their mRNA transcripts, suggested that Cx43 might be involved in maintenance of mitochondrial function or integrity in adipocytes.

**Figure 4:**
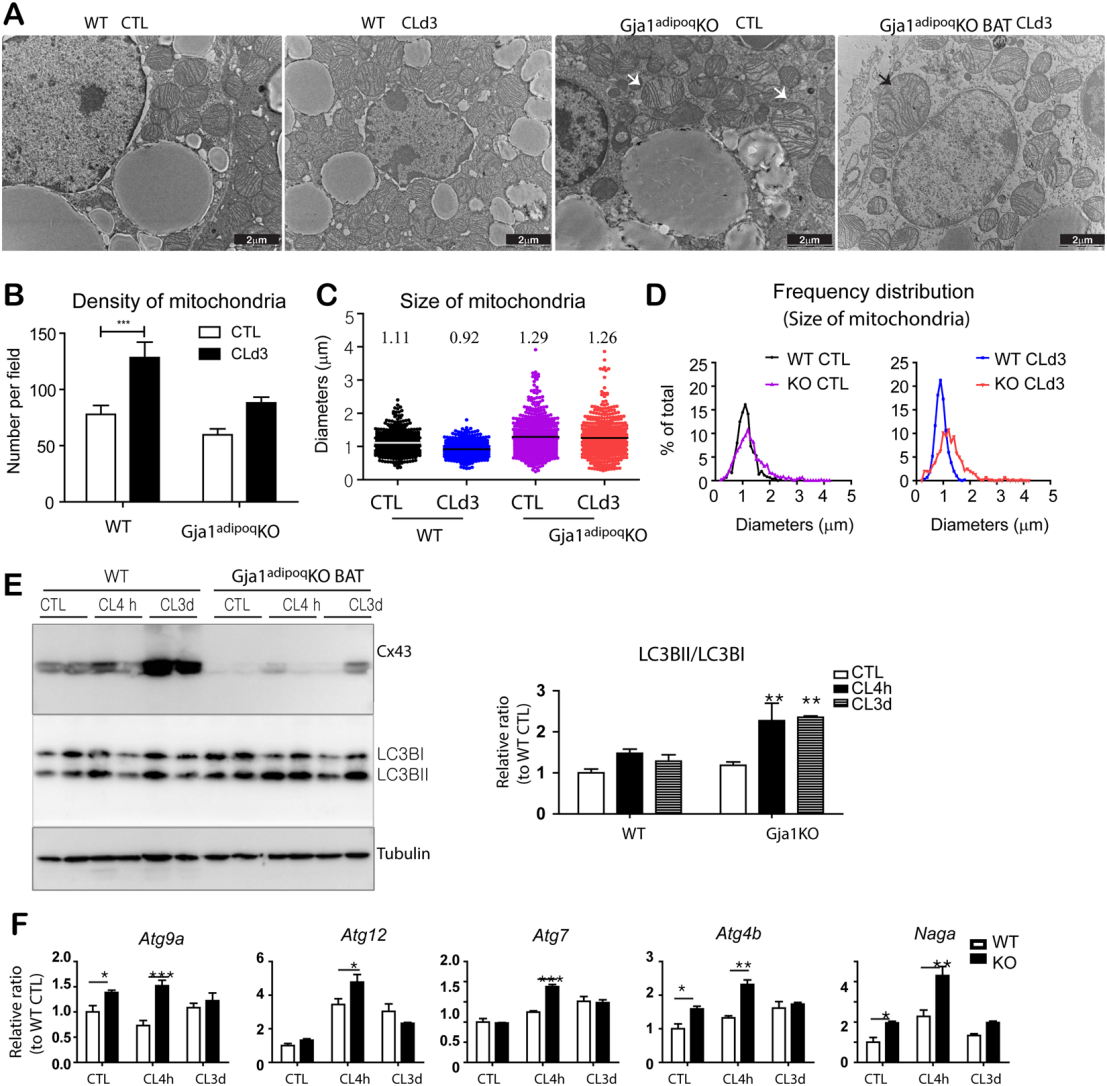
Adipose specific *Gja1* KO generates abnormal mitochondria and increase autophagy in BAT. (**A**) Representative electron micrographs of BAT from WT and *Gja1* ^*adipoq*^KO mice treated with CL for 3 days and untreated controls. (**B**) Mitochondrion density in electron micrographs (mean ± SEM; n = 3 per condition). (**C**) Mitochondrion size in electron micrographs. Mean values were indicated above the scatter plots. (**D**) Distribution analysis of mitochondrion size. (**E**) Immunoblot analysis of LC3I/LC3BII ratio in BAT of *Gja1* ^*adipoq*^KO mice and WT controls treated with CL up to 3 days. Two-way ANOVA revealed significant main effects of *Gja1* expression on LC3BII/LC3BI ratio (p = 0.0053).(mean ± SEM; n = 3 per condition). (**F**) qPCR analysis of autophagy markers in BAT of mice treated with CL for up to 3 days. Two-way ANOVA revealed significant main effects of *Gja1* expression in mRNA levels (*Atg9a*: p = 0.0001, *Atg7*: p = 0.0041, *Atg4b*: p = 0.017, *Naga*: p = 0.0001). (mean ± SEM; n = 3 per condition, *p < 0.01, **p < 0.05, ***p < 0.001). (Immunoblots accompanied by size markers in Fig. S9).

Electron microscopy of BAT demonstrated reduced mitochondrial density and the appearance of abnormal mitochondria in *Gja1* ^*adipoq*^KO mice (Fig. 4A,B). Analysis of the size distribution of mitochondria under basal conditions (22 °C) and following CL stimulation demonstrated the appearance of a population of large mitochondria, some as large as 4μm, that were never observed in wild type (Fig. 4A–D). Frequency of swollen/enlarged mitochondria (i.e. >1.5μ) was greater in the BAT of *Gja1* ^*adipoq*^KO mice (Fig. 4D: chi square = 157; p < 0.00001).

Because the impaired mitochondrial integrity can induce compensatory responses and cell death signals, we examined autophagy in *Gja1* ^*adipoq*^KO mice. An immunoblot analysis of the autophagy marker LC3b suggested that autophagy in BAT was increased in the *Gja1* ^*adipoq*^KO mice compared to that in WT controls after CL treatment (Fig. 4E). Additionally, a qPCR analysis indicated the upregulation of genes involved in autophagy (*Atg9a*, *Atg12*, *Atg7*, *Atg4b*, and *Naga*) in BAT after 4 h of CL treatment (Fig. 4F).

### *In vitro* downregulation of Cx43 reduces mitochondrial content and increases oxidative stress in adipocytes

**Figure 5:**
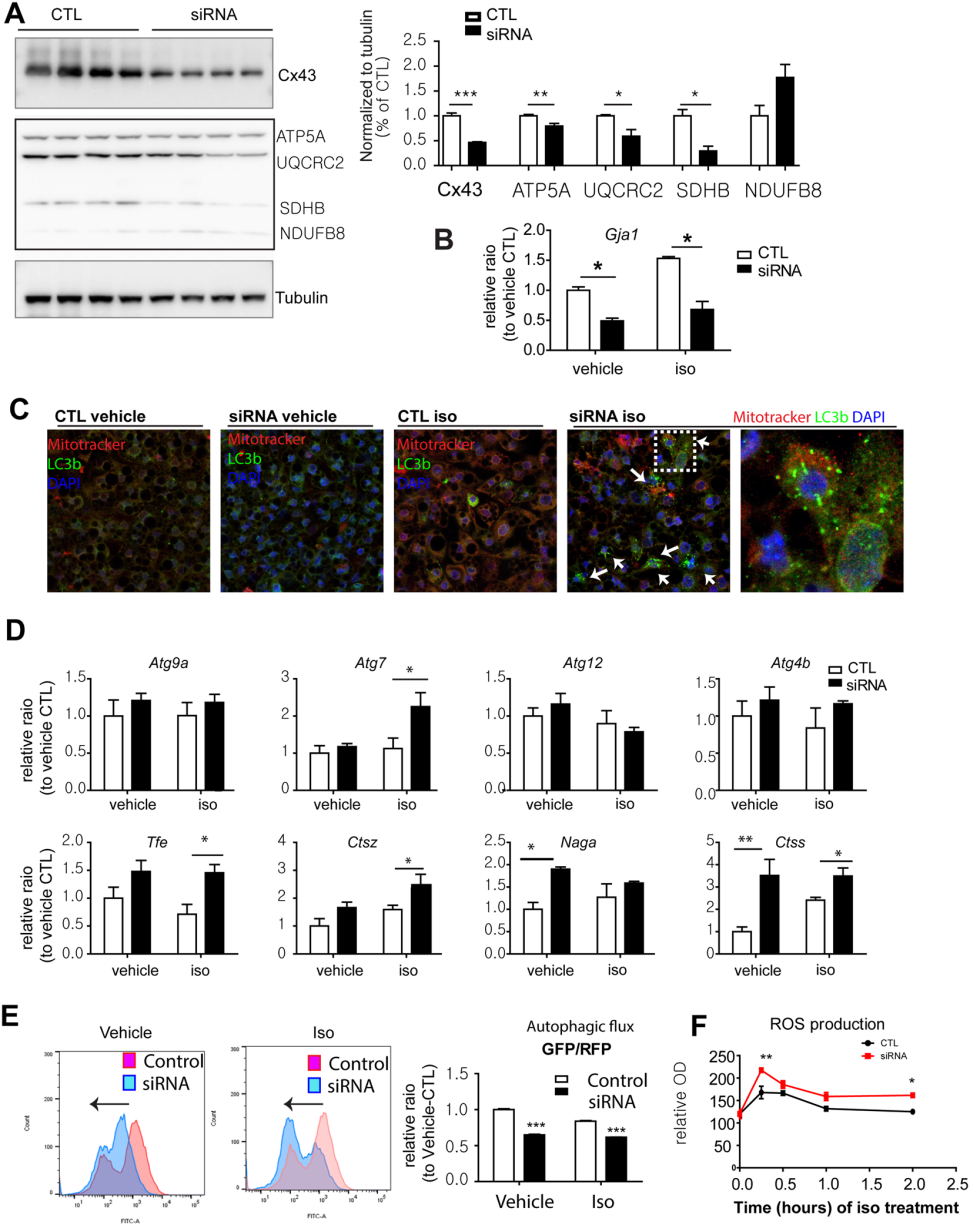
*In vitro* knockdown of Cx43 increases autophagy and PKA-dependent ROS generation. (**A–F**). Effect of knockdown of Cx43 in adipocytes differentiated from C3H10T1/2 cells treated with siRNA targeting *Gja1* and scramble controls. (**A**) Immunoblot analysis of Cx43 and mitochondrial proteins involved in oxidative phosphorylation (mean ± SEM; t-test, n = 4, *p < 0.05, **p < 0.01, ***p < 0.001). (**B**) qPCR analysis of *Gja1* expression (mean ± SEM; t-test, n = 3, *p < 0.05). (**C**) Confocal microscopic images of LC3B and MitoTracker after 4hr of isoproterenol treatment (10μM). (**D**) qPCR analysis of expression of autophagy related genes. Two-way ANOVA revealed significant main effects of Gja1 expression in mRNA levels (*Atg9a*: p = 0.0001, *Atg7*: p = 0.0041, *Atg4b*: p = 0.017, *Naga*: p = 0.0001). Significant differences between controls and siRNA knockdown were determined by post-hoc pairwise comparison with Bonferroni correction (mean ± SEM; n = 4 per condition). (**E**) Flow cytometric analysis of autophagic flux in adipocytes differentiated from C3H10T1/2 cells overexpress eGFP-mCherry-LC3, transfected with siRNA targeting *Gja* or scramble control. The adipocytes were treated with isoproterenol or vehicle for 2hr before analysis. GFP/RFP ratio represents autophagic flux (mean ± SEM; t-test, n = 3, ***p < 0.001). (**F**) ROS generation during the course of isoproterenol treatment for up to 2 hr measured by H2-DCF fluorescence. (Immunoblots accompanied by size markers in Fig. S10).

Because mitochondrial damage was observed in *Gja1* ^*adipoq*^KO mice, we investigated the potential protective roles of Cx43 in mitochondrial homeostasis in adipocytes differentiated from C3H10T1/2 cells using siRNA knockdown of Cx43. Treatment of adipocytes with siRNA reduced Cx43 mRNA and protein by more than 50%, which were maintained for 4 days after siRNA transfection (Fig. 5A and B). Consistent with knockout *in vivo*, knockdown of Cx43 (*Gja1*) significantly reduced expression of components of complex II (SDHB), III (UQCRC2), and V (ATP5A), but not I (NDUFB8) (Fig. 5A).

Immunostaining for LC3B puncta indicated a greater autophagic response to isoproterenol in Cx43 (*Gja1)*-knockdown adipocytes (Fig. 5C). Double label staining with LC3B and the mitochondrial dye, MitoTracker^®^, indicated a close association between these two stains, suggesting an increase in mitophagy (Fig. 5C). Moreover, the suppression of Cx43 expression in adipocytes increased the expression of genes involved in autophagy (*Atg7*, *Tfe*, *Ctsz*, and *Ctss*) after isoproterenol treatment in cells treated with siRNA to knock down *Gja1* (Fig. 5D). Ratio of LC3BII to LC3BI was also increased by siRNA, and treatment with chloroquine (an inhibitor of lysosomal degradation) further increased accumulation of LC3BII compared to controls (Fig. S2C). Next, autophagic flux was measured using an LC3 tandemly tagged with fluorescent proteins that detect lysosomal degradation^14^ in C3H10T1/2 cells. This system expresses a chimeric LC3 fused with eGFP and mCherry, thus pH sensitive decrease in GFP intensity over RFP intensity indicates autolysosome formation. As shown in Fig. 5E, *Gja1* knockdown with siRNA increased autophagic flux, indicated by a decrease in the ratio between the intensities of green and red fluorescence under basal and isoproterenol-activated conditions (Fig. 5E).

To further investigate the protective roles of Cx43 against mitochondrial oxidative stress, we monitored ROS generation and the membrane potential of mitochondria with Cx43 inhibition. β3-adrenergic stimulation with isoproterenol treatment increased ROS levels over time (Fig. 5F). *Gja1* knockdown using siRNA accelerated ROS generation after isoproterenol treatment, and this difference was sustained for 2 h after isoproterenol treatment. The results indicate that Cx43 contributes to the maintenance of mitochondrial integrity and metabolic activity by reducing membrane potential and decreasing ROS generation.

To examine the effect of Cx43 expression in adipocytes, we overexpressed *Gja1* in C3H10T1/2 cells and differentiated them into adipocytes. Endogenous Cx43 was detected in empty-vector control treatments of differentiated C3H10T1/2 cells, and *Gja1* overexpression increased the abundance of Cx43 by 3-fold (Fig. S1). *Gja1* overexpression did not increase the abundance of mitochondrial proteins in adipocytes (Fig. S1). Although *Ppargc1a* expression was induced in *Gja1*-overexpressing cells, the mRNA levels of other mitochondrial proteins (*Cox8b* and *Mcad*) did not change with *Gja1* overexpression. In addition, the expression of brown adipocyte markers such as *Elovl3* and *Cidea* was not affected by *Gja1* overexpression (Fig. S2).

### Adipose-specific *Gja1* knockout produces defective metabolic phenotypes

**Figure 6:**
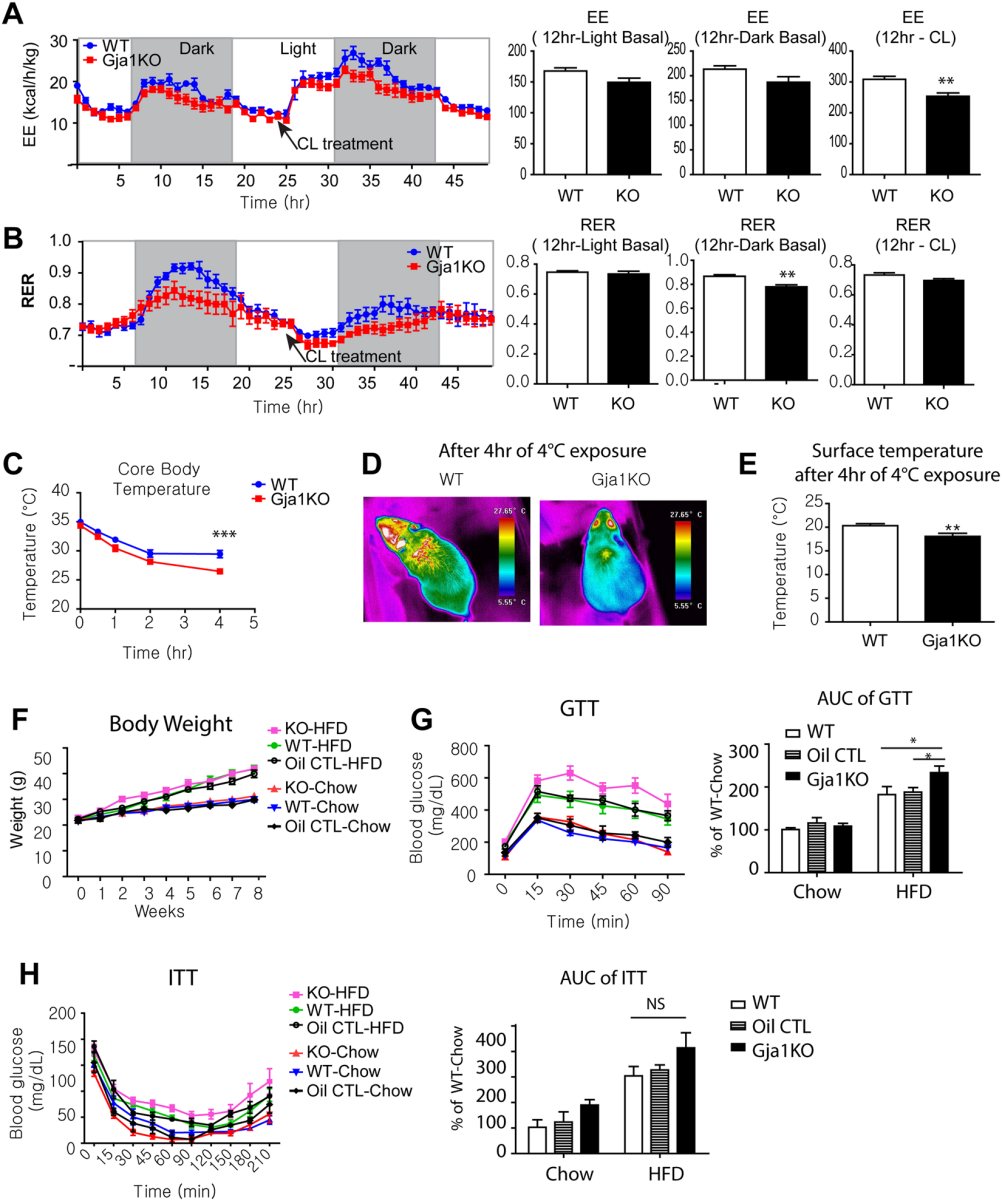
Adipose specific *Gja1* KO manifested reduction in CL-induced energy expenditure, cold intolerance and insulin resistance after a high fat diet challenge. (**A,B**) Indirect calorimetry analysis. Energy expenditure (EE) (**A**) and energy exchange ratio (RER) (**B**) are shown. Arrows indicate the time of CL injection. mean ± SEM, n = 6 per group). (**C–E**) Analysis of body temperature: core body temperature (**C**), thermal images (**D**), surface temperature of region of iBAT (**E**) in WT and *Gja1* ^*adipoq*^KO mice exposed at 22 °C and 4 °C for up to 4 hr (n = 4 per group). (**F–H**) Measurement of body weight (**F**), glucose tolerance test (GTT) (**G**), and insulin tolerance test (ITT) (**H**) in WT (tamoxifen-treated WT/*Gja* ^fl/fl^), Oil-CTL (oil-treated aCre/*Gja* ^fl/fl^) and *Gja1* ^*adipoq*^KO (tamoxifen-treated aCre/*Gja* ^fl/fl^) mice fed with chow or high fat diet (HFD) for 8 weeks (n = 6 per group). Two-way ANOVA revealed significant main effects of genotype (GTT: p = 0.029, ITT: p = 0.047) and diet (GTT: p = 0.001, ITT: p = 0.0003) on area under the curve of GTT and ITT plots. Significant differences between WT and *Gja1* ^*adipoq*^KO were determined by post-hoc pairwise comparison with Bonferroni correction (mean ± SEM; *p < 0.05, NS = non-significant).

Next, we investigated the metabolic phenotype of *Gja1* ^*adipoq*^KO mice by indirect calorimetry (Fig. 6A,B). As expected based on the reduced mitochondrial activity of BAT in *Gja1* ^*adipoq*^KO mice, energy expenditure of *Gja1* ^*adipoq*^KO mice was slightly reduced under basal conditions compared to WT mice. Whereas the immediate thermogenic response to injection of CL316,243 was similar in both genotypes, the sustained response over 12 hrs were significantly lower in *Gja1* ^*adipoq*^KO mice (Fig. 6A). Interestingly, the respiratory exchange ratio was lower in *Gja1* ^*adipoq*^KO mice (Fig. 6B), indicating a preference for free fatty acid over glucose, and their overall metabolic flexibility was defective in *Gja1* ^*adipoq*^KO mice.

When challenged with acute cold stress, *Gja1* ^*adipoq*^KO mice exhibited greater reduction in core body temperature and lower surface temperature over the interscapular BAT (Fig. 6C–E). *Gja1* ^*adipoq*^KO mice gained body weight similarly to WT mice during 8 weeks of high fat diet feeding (Fig. 6F). However, *Gja1* ^*adipoq*^KO mice after 8 weeks of high fat diet feeding manifested insulin resistance, as measured by glucose tolerance tests (Fig. 6G).

## Discussion

The current study confirms that Cx43 is strongly upregulated by direct β3-adrenergic receptor stimulation in adipose tissue^7^ and further demonstrates that significant fraction of Cx43 is targeted to mitochondria in brown adipocytes where it plays an essential role in the maintenance of mitochondrial integrity. It is generally accepted that a major function of Cx43 is to form intercellular gap junction to mediate cell-to-cell communication by allowing the passage of ions and small molecules, such as cAMP^5, 15^. In this regard, recent work has suggested a critical role of Cx43 in coupling of cAMP between adipocytes during beiging of WAT in response to cold stress^7^. Although propagation of the sympathetic nerve signaling through gap junctions is required to facilitate remodelling of WAT with sparse innervation, it does not appear to be critical in BAT with relatively dense innervation^16^. However, the expression levels of Cx43 is much higher in BAT compared to WAT, thus we attempted to investigate a role of Cx43 in BAT metabolism apart from gap junctional communication.

To bypass impact of gap junctions in the propagation of sympathetic activation through cAMP coupling, we pharmacologically induced Cx43 upregulation via CL treatment, which can be uniformly effective in adipocytes that express β3-adrenergic receptors, instead of using neural activation. In this model, we confirmed that PKA downstream signalling was similarly activated in *Gja1* ^*adipoq*^KO and WT mice. We established that Cx43 was abundant in mitochondrial fraction as well as plasma membranes in adipose tissue, and demonstrated that the abundance of Cx43 on the plasma membrane was not significantly changed after CL treatment, yet its mitochondrial localization was nearly tripled in brown adipocytes. It is likely that nascent Cx43 is targeted to mitochondria during ADRB3 stimulation. In support, previous work has demonstrated that Cx43 can be sorted to the inner mitochondrial membrane through heat shock protein 90-mediated TOM pathways.^8^ The genetic deletion of Cx43 compromised the maintenance of mitochondrial integrity and upregulated autophagy in BAT, suggesting that the translocation of Cx43 to mitochondria is a part of the protective mechanism of mitochondria against increased metabolic activity in response to β3-adrenergic stimulation.

We confirmed that β3-adrenergic stimulation increases ROS generation in cultured adipocytes and demonstrated that the inhibition of Cx43 further upregulated production of ROS and membrane potential^17^. It is presently unclear how mitochondrial Cx43 alters membrane potential and ROS generation. Previous work has reported that Cx43 can be translocated into the inner mitochondrial membrane through the heat shock protein 90-dependent translocase of the outer membrane pathway^8^. In general, several factors that increase ROS generation include a high proton gradient, high NADH/NAD^+^ and CoQH_2_/CoQ ratios, and a low local O_2_ concentration^17^. Thus, we speculate that Cx43 in the mitochondrial membrane could modulate ion fluxes or redox status, and thereby contribute to regulating the membrane potential. Whether mitochondrial Cx43 directly interacts with mitochondrial ion channel proteins to reduce membrane potential and supress ROS production is an important question for future studies. We showed that downregulation of Cx43 influenced expression levels of Atg9a, Atg7, Atg12 and Atg4b. Although the transcriptional control of Atg gene expression was not examined in this study, it would be informative to further investigate effects of Cx43 on the regulation of transcription factors related to autophagy induction.

While diverse roles of gap junctions have been reported^6, 15^, it has been shown that gap junctions play critical roles in adipogenesis^18^ and lipid metabolism^4^ of adipocytes. For instance, recent work demonstrated that an increase in calcium levels after treatment with capsaicin is mediated by Cx43 and promotes lipolysis in visceral adipose tissue^4^. It would be informative to study role of Cx43 in other subcellular location in adipocytes. In addition to Cx43 in mitochondria, we cannot exclude the possibility that Cx43 in other subcellular compartments may have roles in adipocyte function and mitochondrial homeostasis. In this regard, a recent study demonstrated that Cx43 exerts anti-autophagic effects by interacting with autophagy signalling mediators in the plasma membrane^19^.

Although the current study focused on Cx43 in BAT, the role of mitochondrial Cx43 in WAT deserves further investigation. We demonstrated that WAT showed increased Cx43 expression during β3-adrenergic remodelling. In addition, cultured white adipocytes that did not express UCP1 also showed increased ROS generation and autophagy after the inhibition of Cx43 expression. Thus, Cx43 might be a general protective mechanism against mitochondrial oxidative stress in adipocytes. We suggest that a lack of Cx43 in white adipocytes could increase their sensitivity to inflammation and cell-death induced by lipolysis. Our previous work suggested that β3-adrenergic stimulation increases the apoptotic death of adipocytes in gonadal adipose tissue^16, 20, 21^ and it is possible that the upregulation of Cx43 in adipocytes and its targeting into mitochondria are required for resolving the transient inflammatory response induced by β3-adrenergic stimulation. It remains to be determined whether the overexpression of Cx43 in WAT might protect against oxidative stress and lipolysis-induced cell death during β3-adrenergic stimulation.

The activation of mitochondrial metabolism in BAT can increase energy expenditure to control energy homeostasis^2^. In general, a decrease in mitochondrial activity is involved in the pathogenesis of metabolic disorders and the aging process^22^. Furthermore, a failure to counteract the oxidative stress generated from mitochondrial respiration can initiate cell death processes and contribute to the development of metabolic disease^22^. Thus, understanding the adaptive responses of mitochondria to metabolic challenges and the coordinated upregulation of oxidant defence mechanisms to maintain mitochondrial quality is central to combating metabolic disease. The adaptation of mitochondria to catabolic stimuli, combined with the induction of protective mechanisms against oxidative stress, may represent a strategy that could be developed for therapeutic applications, and mitochondrial gap junctional proteins in adipocytes may be pharmaceutical targets for combating metabolic disease.

## Methods

### Animals

All animal experiments were conducted in strict compliance with the guidelines for humane care and use of laboratory animals as specified by the Ministry of Food and Drug Safety. All animal protocols were approved by the Institutional Animal Care and Use Committees at Yonsei University. C57BL/6 mice (5–6 wk old, male) were purchased from Orient Bio (Gyeonggi-Do, South Korea). *Gja1* ^flox/flox^ mice (stock #008039: B6.129S7-Gja1tm1Dlg/J) and Adipoq-CreER mice (Stock #024671: B6.129-Tg (Adipoq-cre/Esr1*)1Evdr/J) were purchased from the Jackson Laboratory and crossed to produce inducible adipose tissue specific *Gja1* (the gene for Cx43) knockout (Adipoq-CreER/*Gja1* ^flox/flox^) mice and *Gja1* ^flox/flox^ mice without CreER for control wild type mice. Genotyping was performed as described previously^23, 24^. Mice were housed at 22 °C and maintained on a 12-h light/12-h dark cycle with free access to standard chow diet and water at all time. For Cre recombination, double transgenic mice (Adipoq-CreER/*Gja1* ^flox/flox^ mice, 5 week old) and wild type controls (*Gja1* ^flox/flox^ mice without CreER) were treated with tamoxifen dissolved in sunflower oil (Sigma, 75 mg/kg) by oral gavage on each of 5 consecutive days. Also, we included vehicle (oil)-treated control groups to confirm specific induction of Cre recombinase activity upon tamoxifen treatment in adipose tissue. Experiments were started 10 days after the last dose of tamoxifen. For β3 adrenergic receptor stimulation, mice were treated with CL316,243 (Sigma, St. Louis, MO) (1 mg/kg/day) by intraperitoneal injection for up to 5 days. For the high fat diet (HFD) experiments, 60% fat diet (D12492, Central Lab. Animal Inc.) was introduced at 7 weeks of age and continued for 8 weeks.

For exposure to cold stress, mice were single caged in cold room (4 °C) and body temperature was measured by thermometers (New Homeothermic Blanket Monitoring System, Harvard Apparatus, Canada) with rectal probe and images were taken with Infra-red camera (CX320, COX, South Korea).

For glucose tolerance test and insulin tolerance test, mice were given D-glucose (2 mg/ml, sigma) and insulin (0.75U/kg, Sigma) by intraperitoneal injection respectively, and glucose concentrations were measured at indicated time points.

Metabolic measurements were obtained using indirect calorimetry system (PhenoMaster, TSE system, Bad Homburg, Germany). The mice were acclimatized to the cages for 2 days, and O_2_ consumption (VO_2_), CO_2_ production (VCO_2_), food intake and locomotor activity were monitored for 3 days while food and water were provided ad libitum.

## Cell Culture

For adipogenic differentiation, C3H10T1/2 mouse embryonic fibroblasts (American Type Culture Collection (ATCC), Manassas, VA) were cultured to confluence in growth medium (DMEM supplemented with 10% fetal bovine serum (FBS) and 1% penicillin/streptomycin at 37 °C in a humidified atmosphere with 5% CO_2_ and then exposed to bone morphogenetic protein 4 (20 ng/ml; R&D Systems, Minneapolis, MN) followed by exposure to differentiation medium (DMEM supplemented with 10% FBS, 1% P/S, 2.5 mM isobutylmethylxanthine, 1 μM dexamethasone, and 1 μg/ml insulin for 3 days and then maintained in medium containing 1 μg/ml insulin for 4 d. To induce β-adrenergic stimulation, differentiated adipocytes were incubated in growth medium for 24 hours, and treated with isoproterenol (10μM, Sigma). For inhibition of autophagy, choloroquine (an inhibitor of late phase (lysosomal degradation), 50μM, Sigma) and 3-methyl adenine (3-MA, an inhibitor of early phase, 10 mM, Sigma) were used. Adipocytes were treated with inhibitors for 30 min before isoproterenol treatment.

For *Gja1* knockdown, siRNA targeting *Gja1* (Sigma, CAT. #EMU006781) were transfected into adipocytes differentiated from C3H10T1/2, using lipofectamine (ThermoFisher).

To prepare Cx43 overexpressing preadipocytes, C3H10T1/2 cells were infected with pLPCX-CX43-IRES-GFP (Addgene, plasmid #65433, a gift from Trond Aasen)^25^ by using retrovirus infection described previously. Briefly, for retroviral infections, viral constructs were transfected into phoenix cells using lipofectamin2000 (ThermoFisher). Supernatants were collected, supplemented with 8 mg/ml hexadimethrine bromide (Sigma) and then exposed to the cultures. Infected preadipocytes were selected with 2 μg/ml of puromycin for 1 week.

Alternatively, differenitated adipocytes from C3H10T1/2 cells were infected wth pLVX-EIP-hCx43 by using lentivirus infection. Briefly, human Cx43 cDNA was amplified with PCR using 5′-GC TCT AGA ACC ATG GGT GAC TGG AGC GC-3′ and 5′-GC TCT AGA CTA GAT CTC CAG GTC ATC AGG C-3′ and inserted at XbaI site of pLVX-EF1a-IRES-Puro (Clontech), generating pLVX-EIP-hCx43. To produce lentivirus expressing hCx43, pLVX-EIP-hCx43, psPAX2 (Addgene, plasmid #12260), and, pMD2.G (Addgene, plasmid #12259) were mixed at 4:3:1 and transfected into HEK293T cells with iN-fect (iNtRON Biotechnology, Seongnam, Korea) overnight. The conditioned medium was harvested ﻿after 48 hours and cleared with filtration through 0.44 nm pore before use.

For autophagic flux analysis, C3H10T1/2 cells were infected with pBABE-puro mCherry-EGFP-LC3B^14^ (Addgene, plasmid #22418, a gift from Jayanta Debnath) by using retrovirus infection as described above. For autophagic flux analysis, adipocytes differentiated from C3H10T1/2 cells that express mCherry-EGFP-LC3B was analyzed by using BD FACSAria III (BD Biosciences, San Jose, CA, USA). Raw data were processed using FlowJo software (Tree Star, Ashland, OR, USA).

To measure ROS production, adipocytes differentiated from C3H10T1/2 cells were treated with 10μM isoproterenol for indicated time and then exposed to 20μM H2DCFDA (Thermo Fisher Scientific) for 30 min. 40 nM H_2_O_2_ (Sigma) was used as a positive control. Fluorescence intensity was determined by Tecan microplate reader with 485 nm excitation and 535 nm emission. Alternatively, cellls were treated with CellROX Green Reagent (Thermo Fisher) for 30 min for ROS detection by fluorescence microscopy.

### Subcellular fractionation

Subcellular fractionation was performed as described previously^26, 27^. Briefly, from tissue homogenates in fractionation buffer (containing 3 mM HEPES (pH7.4), 210 mM mannitol, 70 mM sucrose and 0.2 mM EDTA), cells and debris pellets were removed after centrifugation at 500 × g for 10 min. After centrifugation of the supernatant at 10,000 × g for 10 min, pellets containing mitochondria were collected and supernatant containing non-mitochondrial fraction wase centrifuged at 95000 × g for 2 h at 4 °C to obtain the plasma membrane fraction (pellet) and cytosolic fraction (supernatants). For the further purification, mitochondrial fractions were separated by differential centrifugation in sucrose gradient (30–60% w/v) at 100,000 × g. For outer mitochondrial membrane protein extraction, crude mitochondrial fractions were treated at 4 °C for 10 min or 30 min with PBS containing 0.05% trypsin (Sigma). Resulting fractions were subjected to western blot analysis.

### Western blot

Protein extracts were prepared as previously described^29^. Western blot analysis was performed using primary antibodies against UCP1 (mouse, Abcam; or rabbit Alpha Diagnostic International), Cx43 (rabbit, Sigma), COX IV (rabbit, Cell Signaling), Na^+^/K^+^ ATPase (rabbit, Cell Signaling), GAPDH (mouse, Bethyl), Total OXPHOS Rodent WB Antibody Cocktail (mouse, Abcam), p-HSL (rabbit, Cell signaling), HSL (rabbit, Cell Signaling), LC3b (rabbit, Cell signaling), caveolin1 (rabbit, Sigma), cabeolin 3 (rabbit, Sigma), Tom22 (Cell Signaling, rabbit) and a/b tubulin (rabbit, Cell Signaling) and secondary anti-mouse, and anti-rabbit horseradish peroxidase antibodies (Cell Signaling Technology, Danvers, MA), as described previously^28^. The blots were visualized with SuperSignal West Dura Substrate (Pierce-Invitrogen).

### qPCR

For quantitative PCR analysis, RNA was extracted using TRIzol reagent (Invitrogen) and converted into cDNA by using high-capacity cDNA synthesis kit (Applied Biosystems, Waltham, MA). Quantitative PCR was performed using SYBR Green Supermix (Bio-Rad) and CFX Real-time PCR System (Bio-Rad) for 45 cycles, and the fold change for all the samples was calculated by the comparative cycle-threshold (Ct) method (i.e., 2− Δ Δ Ct method). Peptidylprolyl isomerase A was used as the housekeeping gene for mRNA expression analysis. cDNA was amplified using the primers listed in Table S1 or described previously^29^.

### Immunohistochemistry

Adipose tissue was processed for histological sections, and 5 μm-thick paraffin sections were subjected to immunohistochemical analysis, as previously described^30^.

### Transmission electron microscopy (TEM)

Small pieces of minced BAT (1~2mm^3^) were BAT were immersed with 2% paraformaldehyde and 2.5% glutaraldehyde in 0.1 M phosphate buffer (pH 7.4). The tissue was removed and stored in the same fresh fixative overnight at 4 °C. Tissues were washed, post-fixed in 1% osmium tetroxide for 2 h, and dehydrated through an ascending series of ethanol, propylene oxide, and embedded in Epon mixture (Oken Shoji, Japan). Thin sections (70 nm) were made using Leica EM UC7 ultramicrotome (Leica Microsystems, Wetzlar, Germany), mounted on 200 mesh copper grids, stained with 2% uranyl acetate and 1% lead citrate for 5 min each, and observed under a Hitachi H-7650 transmission electron microscope (Hitachi, Tokyo, Japan) at the accelerating voltage of 80 kV.

### Statistical analysis

Statistical analyses were performed using GraphPad Prism 5 software (GraphPad Software, La Jolla, CA, USA.). Data are presented as mean ± SEM. Statistical significance between two groups was determined by unpaired t-test or Mann-Whitney test, as appropriate. Comparison among multiple groups was performed using a one-way analysis of variance (ANOVA) or two-way ANOVA, with Bonferroni post hoc tests to determine p values.

## Electronic supplementary material

Supplemental information

## References

[1] Cannon B, Nedergaard J (2004). Brown adipose tissue: function and physiological significance. Physiol. Rev..

[2] Kajimura S, Spiegelman BM, Seale P (2015). Brown and Beige Fat: Physiological Roles beyond Heat Generation. Cell Metab.

[3] Burke S (2014). Adipocytes in both brown and white adipose tissue of adult mice are functionally connected via gap junctions: implications for Chagas disease. Microbes and Infection.

[4] Chen J (2015). Activation of TRPV1 channel by dietary capsaicin improves visceral fat remodeling through connexin43-mediated Ca2+ Influx. Cardiovascular Diabetology.

[5] Evans WH, Martin PE (2002). Gap junctions: structure and function (Review). Molecular membrane biology.

[6] Revel JP, Yee AG, Hudspeth AJ (1971). Gap Junctions between Electrotonically Coupled Cells in Tissue Culture and in Brown Fat. Proceedings of the National Academy of Sciences of the United States of America.

[7] Zhu Y (2016). Connexin 43 Mediates White Adipose Tissue Beiging by Facilitating the Propagation of Sympathetic Neuronal Signals. Cell Metab.

[8] Rodriguez-Sinovas A (2006). Translocation of Connexin 43 to the Inner Mitochondrial Membrane of Cardiomyocytes Through the Heat Shock Protein 90–Dependent TOM Pathway and Its Importance for Cardioprotection. Circulation Research.

[9] Boengler KK (2005). Connexin 43 in cardiomyocyte mitochondria and its increase by ischemic preconditioning. Cardiovascular research.

[10] Boengler K (2012). Mitochondrial connexin 43 impacts on respiratory complex I activity and mitochondrial oxygen consumption. Journal of Cellular and Molecular Medicine.

[11] Cannon B, Nedergaard J (2008). Developmental biology: Neither fat nor flesh. Nature.

[12] Lee YH, Kim SN, Kwon HJ, Granneman JG (2017). Metabolic heterogeneity of activated beige/brite adipocytes in inguinal adipose tissue. Scientific reports.

[13] Solan JL, Lampe PD (2005). Connexin phosphorylation as a regulatory event linked to gap junction channel assembly. Biochim Biophys Acta.

[14] N’Diaye EN (2009). PLIC proteins or ubiquilins regulate autophagy-dependent cell survival during nutrient starvation. EMBO Rep.

[15] Goodenough DA, Goliger JA, Paul DL (1996). Connexins, Connexons, and Intercellular Communication. Annual Review of Biochemistry.

[16] Lee Y-H, Petkova AP, Granneman JG (2013). Identification of an Adipogenic Niche for Adipose Tissue Remodeling and Restoration. Cell Metab.

[17] Mailloux RJ, Harper M-E (2011). Uncoupling proteins and the control of mitochondrial reactive oxygen species production. Free Radical Biology and Medicine.

[18] Yeganeh, A. *et al*. Connexin 43 phosphorylation and degradation are required for adipogenesis. *Biochim Biophys Acta* **1823**, doi:10.1016/j.bbamcr.2012.06.009 (2012). 10.1016/j.bbamcr.2012.06.009 22705883

[19] Bejarano, E. *et al*. Connexins modulate autophagosome biogenesis. *Nat Cell Biol* **16**, 401–414, doi:10.1038/ncb2934, http://www.nature.com/ncb/journal/v16/n5/abs/ncb2934.html#supplementary-information (2014). 10.1038/ncb2934 PMC4008708 24705551

[20] Kwon HJ, Kim SN, Kim YA, Lee YH (2016). The contribution of arachidonate 15-lipoxygenase in tissue macrophages to adipose tissue remodeling. Cell death & disease.

[21] Li P, Zhu Z, Lu Y, Granneman JG (2005). Metabolic and cellular plasticity in white adipose tissue II: role of peroxisome proliferator-activated receptor-α. Am J of Physiol Endocrinol Metab.

[22] Chow J, Rahman J, Achermann JC, Dattani MT, Rahman S (2017). Mitochondrial disease and endocrine dysfunction. Nat Rev Endocrinol.

[23] Mottillo EP (2014). Coupling of lipolysis and de novo lipogenesis in brown, beige, and white adipose tissues during chronic beta3-adrenergic receptor activation. J Lipid Res.

[24] Liao Y, Day KH, Damon DN, Duling BR (2001). Endothelial cell-specific knockout of connexin 43 causes hypotension and bradycardia in mice. Proceedings of the National Academy of Sciences.

[25] Salat-Canela C, Sese M, Peula C, Ramon y Cajal S, Aasen T (2014). Internal translation of the connexin 43 transcript. Cell communication and signaling: CCS.

[26] Wieckowski MR, Giorgi C, Lebiedzinska M, Duszynski J, Pinton P (2009). Isolation of mitochondria-associated membranes and mitochondria from animal tissues and cells. Nat. Protocols.

[27] Chung YW (2015). Targeted disruption of PDE3B, but not PDE3A, protects murine heart from ischemia/reperfusion injury. Proceedings of the National Academy of Sciences.

[28] Kim S-N (2016). Sex differences in sympathetic innervation and browning of white adipose tissue of mice. Biology of sex differences.

[29] Lee Y-H, Kim S-N, Kwon H-J, Maddipati KR, Granneman JG (2016). Adipogenic role of alternatively activated macrophages in β-adrenergic remodeling of white adipose tissue. American Journal of Physiology - Regulatory, Integrative and Comparative Physiology.

[30] Lee Y-H, Petkova AP, Mottillo EP, Granneman JG (2012). *In Vivo* Identification of Bipotential Adipocyte Progenitors Recruited by β3-Adrenoceptor Activation and High-Fat Feeding. Cell Metab.

