# Supplementary material for: Connexin 43 is required for the maintenance of mitochondrial integrity in brown adipose tissue
Source: Sci Rep. 2017 Aug 2;7:7159. doi: 10.1038/s41598-017-07658-y (PMC5540980; doi:10.1038/s41598-017-07658-y)
Supplement: Supplementary file 1 — Supplemental information [file 41598_2017_7658_MOESM1_ESM.pdf]

Title: Connexin 43 is required for the maintenance of mitochondrial integrity in brown adipose tissue.

Author list and affiliations: Sang-Nam Kim [#1], Hyun-Jung Kwon[#1], Seo-Woo Im[1], Yeon-Ho Son[1], Seun Akindehin[1], Young-Suk Jung[2,] Se Jeong Lee[3], Im Joo Rhyu[3], Il Yong Kim[4], Je-Kyoung Seong[4], Jinu Lee[1], Hee-Chan Yoo [1], James G. Granneman[5], Yun-Hee Lee*[1]

[1] College of Pharmacy, Yonsei University, Incheon 21983, South Korea,

[2] College of Pharmacy, Pusan National University, Busan 46241, South Korea,

[3] Department of Anatomy, Korea University College of Medicine, Seoul 02841, South Korea

[4] Laboratory of Developmental Biology and Genomics, College of Veterinary Medicine, Seoul National University, Korea Mouse Phenotyping Center (KMPC), Seoul 08826, South Korea

[5] Center for Integrative Metabolic and Endocrine Research, Wayne State University School of Medicine, Detroit, MI, USA, 48201

[#] These authors contributed equally to this work.

***Corresponding author**

Yun-Hee Lee, PhD
College of Pharmacy, Yonsei University
310 Veritas Hall D, 85 Songdogwahak-ro, Yeonsu-gu, Incheon, 21983, Korea
Tel: 82-32-749-4522
Fax: 82-32-749-4105
e-mail: yunhee.lee@yonsei.ac.kr

Supplemental information

Figure S1. Immunoblot analysis of Cx43 expression in mitochondrial fractions of BAT of (B) mice treated with CL 316,243 and (A) control conditions.

Figure S2. Immunoblot analysis of Cx43 and mitochondrial proteins in BAT of Gja KO mice treated with CL 316,243 and control conditions (related to Figure 3).

Figure S3. *In vitro* knockdown of Cx43 increases autophagy and PKA-dependent ROS generation (supplemental to Figure 5).

Figure S4. *In vitro* overexpression of Cx43 does not affect levels of mitochondrial enzymes.

Figure S5. Graphical summary of potential roles of Cx43 in the maintenance of mitochondrial integrity in brown adipocytes.

Figure S6. Immunoblots accompanied by size markers (used in Figure 1)

Figure S7. Immunoblots accompanied by size markers (used in Figure 2)

Figure S8. Immunoblots accompanied by size markers (used in Figure 3)

Figure S9. Immunoblots accompanied by size markers (used in Figure 4)

Figure S10. Immunoblots accompanied by size markers (used in Figure 5)

Table S1. List of murine primers used for real time qPCR

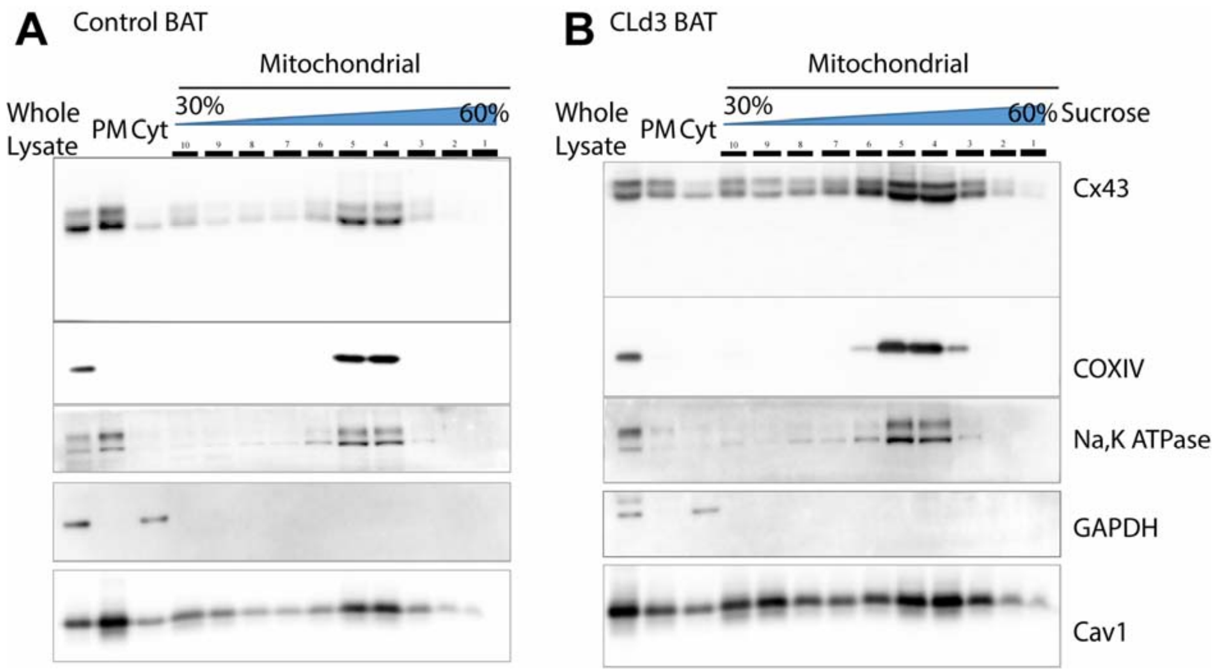

Figure S1. Immunoblot analysis of Cx43 expression in mitochondrial fractions of BAT of (B) mice treated with CL 316,243 and (A) control conditions.

Cx43 expression was determined in subcellular fractions including whole lysate, plasma membrane fraction, cytosolic fraction and mitochondrial fraction obtained by sucrose density gradient as indicated.

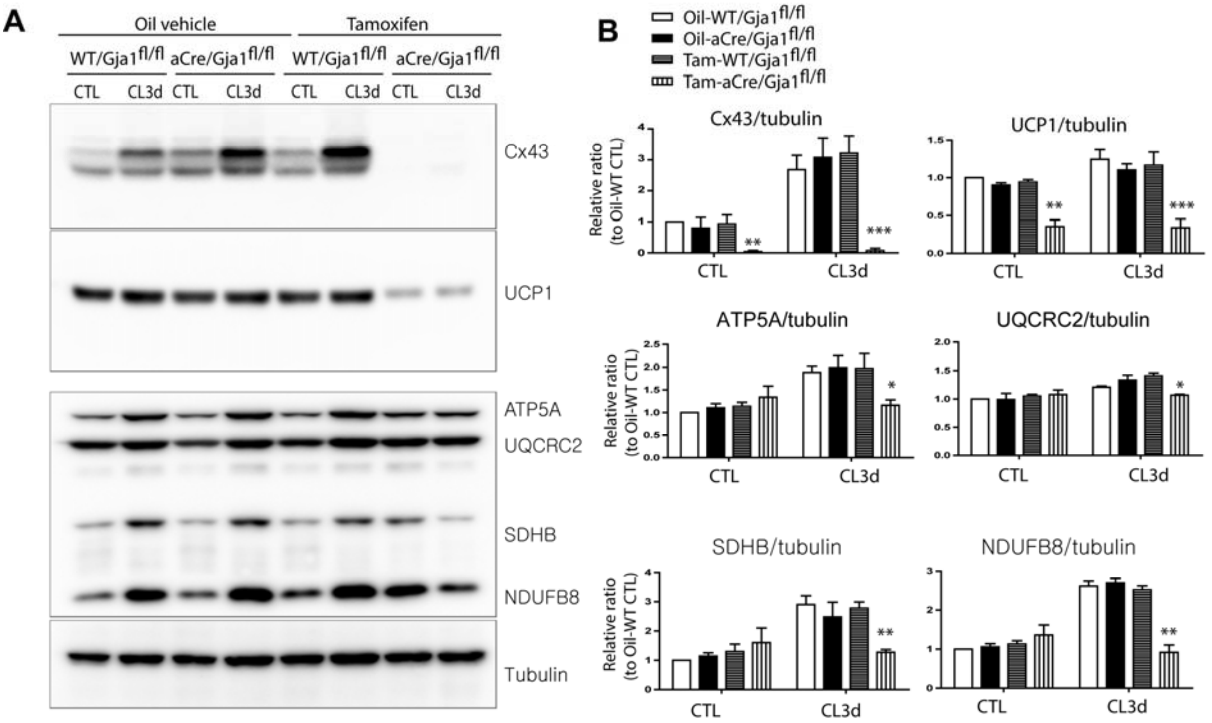

Figure S2. Immunoblot analysis of Cx43 and mitochondrial proteins in BAT of adipocyte-specific Gja KO mice treated with CL 316,243 for 3 days and control conditions. (related to Figure 3)

Significant differences between WT (Tamoxifen-treated WT/Gja1$^{fl/fl}$) and adipocyte-specific Gja1KO (Tamoxifen-treated aCre/Gja1$^{fl/fl}$) were determined by post-hoc pairwise comparison with Bonferroni correction (mean ± SEM; n = 3 per condition, *p<0.01, **p<0.05, ***p<0.001). Oil vehicle-treated groups (oil- aCre/Gja1$^{fl/fl}$, oil-WT/Gja1$^{fl/fl}$) were included, showing no significant effect of tamoxifen treatment on Cx43, UCP1 and mitochondrial proteins involved in oxidative phosphorylation.

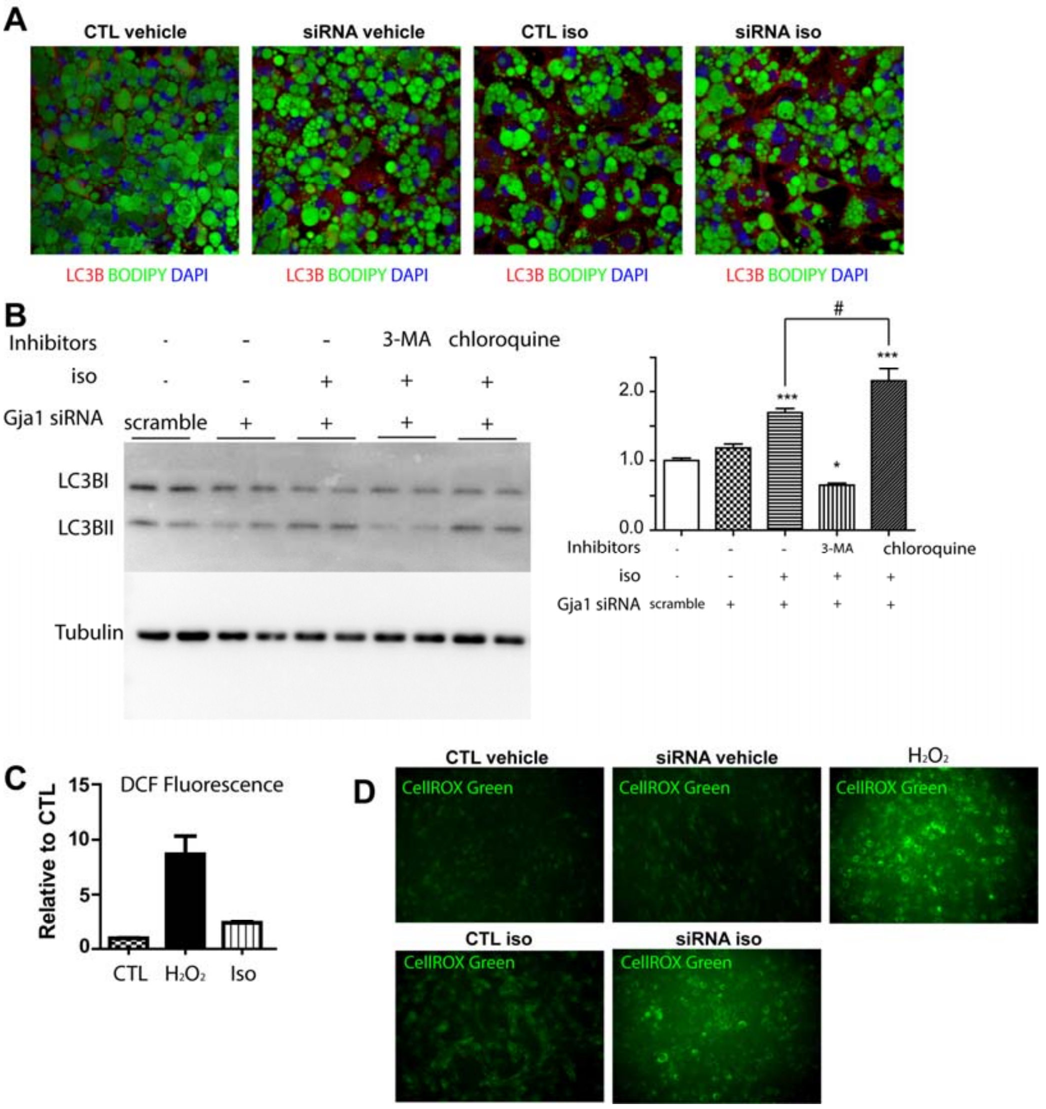

Figure S3. ***In vitro*** **knockdown of Cx43 increases autophagy and PKA-dependent ROS generation (supplemental to Figure 5).**

A. Effect of knockdown of Cx43 in adipocytes differentiated from C3H10T1/2 cells treated with siRNA targeting *Gja1* and scramble controls. Confocal microscopic images of LC3B and BODIPY® 493/503 (4,4-Difluoro-1,3,5,7,8-Pentamethyl-4-Bora-3a,4a-Diaza-s-Indacene) after 4hr of vehicle or isoproterenol treatment (10 µM). B. Immunoblot analysis of LC3I/LC3BII ratio in adipocytes differentiated from C3H10T1/2. Adipocytes were pretreated with inhibitors (3-methyladenine (3-MA, 5 mM), or chloroquine (50 µM)) for 30 min, and then treated with isoproterenol (10µM, 4 hr). Significant differences compared to controls (*$p<0.05$, ***$p<0.001$) and differences between vehicle and chloroquine treatment (#$p<0.05$) were determined by t-test (mean ± SEM; n = 4). C. ROS generation after 30 min of H_2O_2 (40nM) exposure or isoproterenol (10µM) treatment measured by H2-DCF fluorescence. D. Immunofluorescence detection of ROS generation by CellROX green reagent in adipocytes transfected with siRNA or scramble controls, and treated with isoproterenol (10µM), H_2O_2 (40nM) or vehicles for 30min.

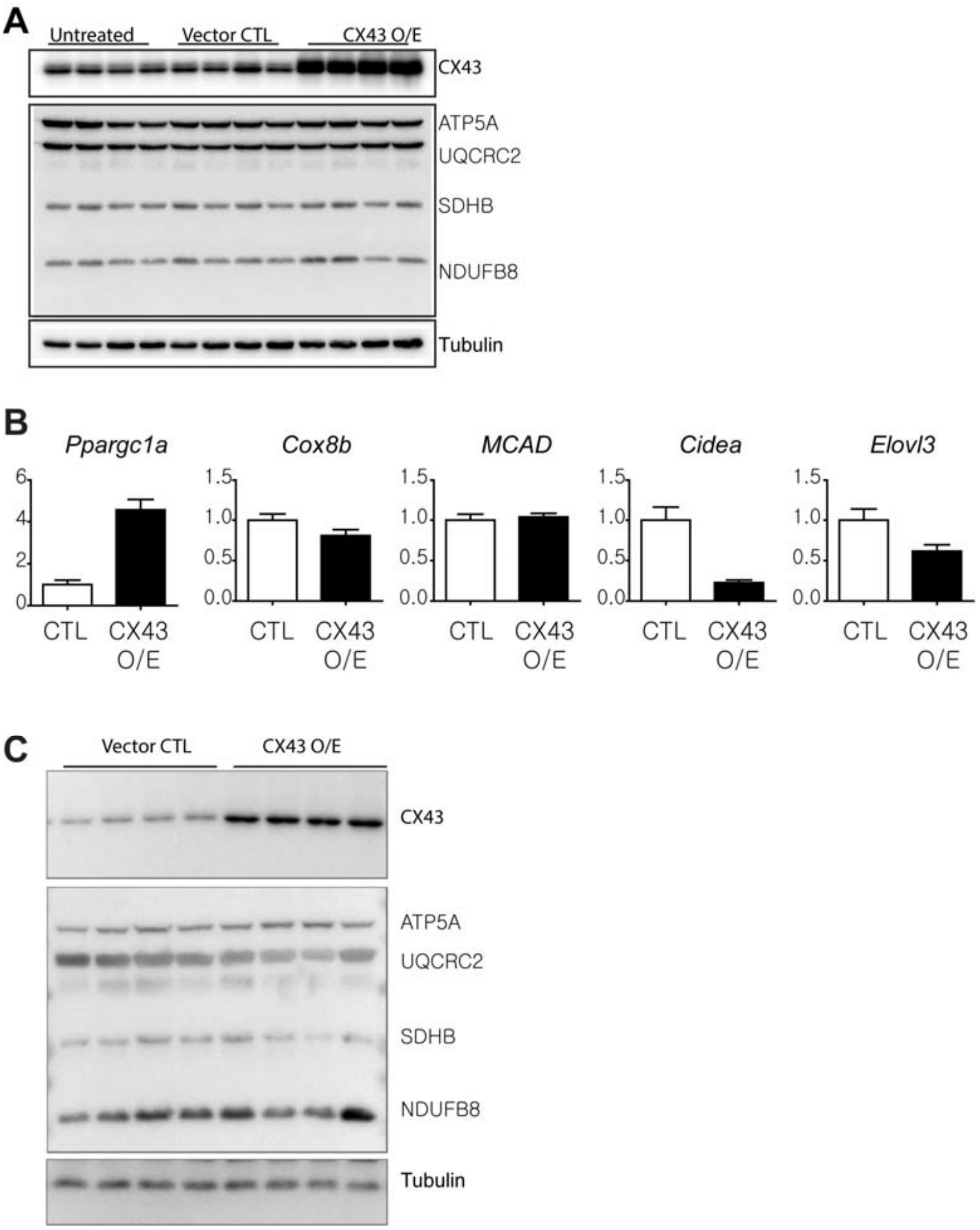

Figure S4. *In vitro* overexpression of Cx43 does not affect levels of mitochondrial enzymes.

A. Immunoblot analysis of Cx43 and mitochondrial proteins involved in oxidative phosphorylation in adipocytes differentiated from C3H10T1/2 cells overexpressing Gja1 and controls. B. qPCR analysis of expression of mitochondrial enzymes and brown adipocyte markers in adipocytes differentiated from C3H10T1/2 cells overexpressing Gja1 and controls. C. Immunoblot analysis of Cx43 and mitochondrial proteins involved in oxidative phosphorylation in adipocytes differentiated from C3H10T1/2 cells that were infected with lentivirus expressing Gja1 after full differentiation and controls.

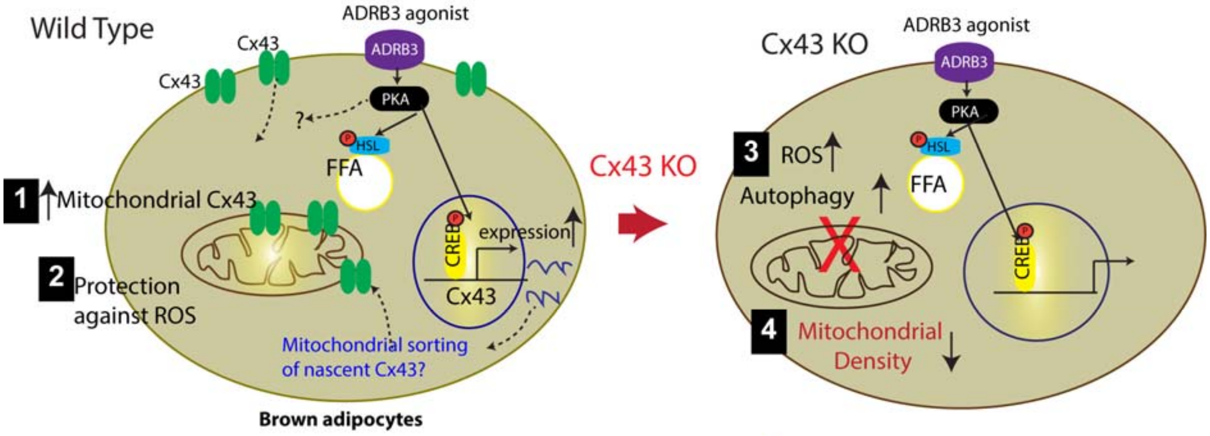

Figure S5. Graphical summary of potential roles of Cx43 in the maintenance of mitochondrial integrity in brown adipocytes.

Data indicate that Cx43 is important in the maintenance of mitochondrial integrity under basal and beta adrenergic stimulation. 1) Mitochondrial targeting of Cx43 upon beta adrenergic stimulation may contribute to 2) protection of mitochondria against oxidative stress during metabolic activation. In addition, data indicate that ADRB3 agonist treatment increased mitochondrial metabolism and ROS generation, and 3) defect in mitochondrial protection caused by Gja1KO accelerated autophagy. 4) We hypothesize that generation of abnormal mitochondria could be a stressful condition that activates autophagy to remove defective mitochondria, consequently resulting in reduction of mitochondrial density.

Figure 1B- Cx43

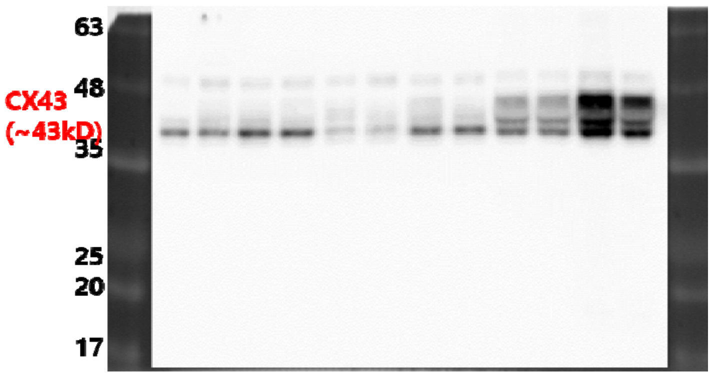

Figure 1B- Tubulin

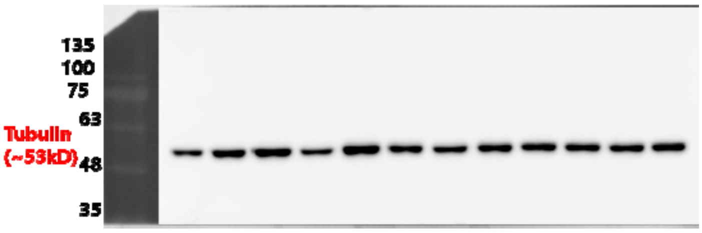

Figure 1C- Cx43

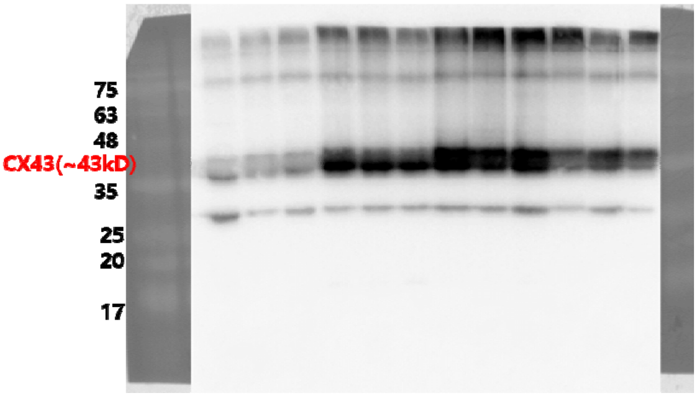

Figure 1C- UCP1

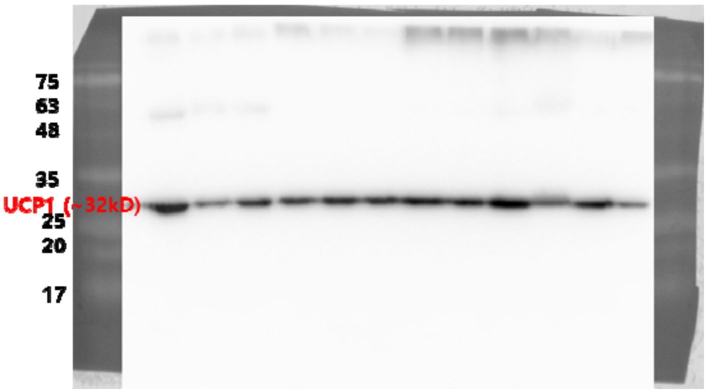

Figure 1C- Tubulin

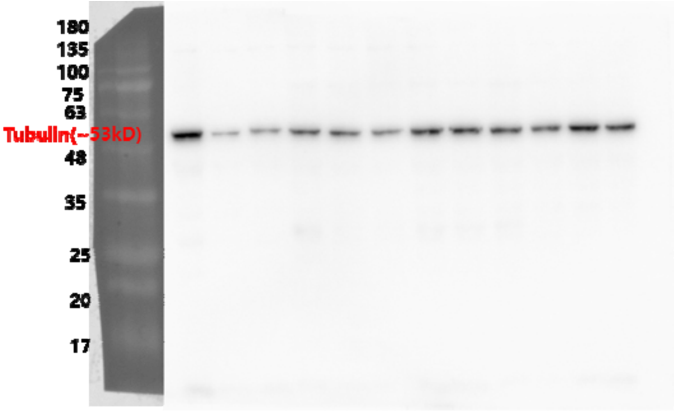

Figure 1D- Cx43

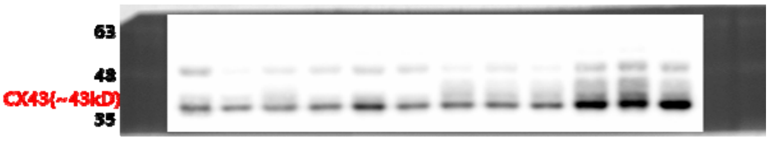

Figure 1D- UCP1

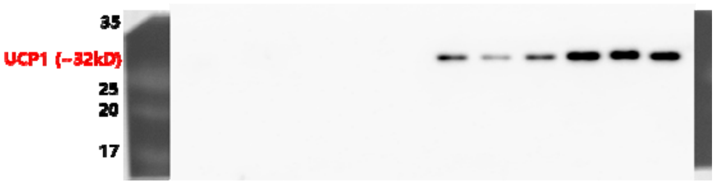

Figure 1D- Tubulin

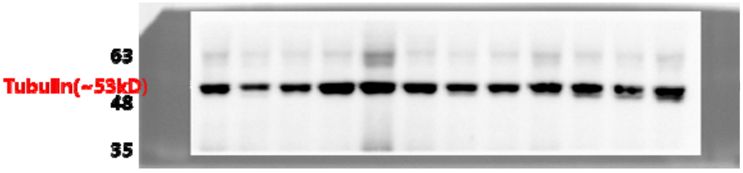

Figure 1E- Cx43

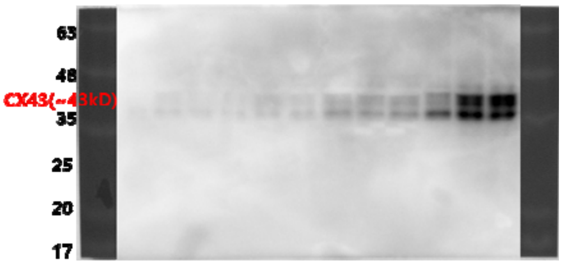

Figure 1E- UCP1

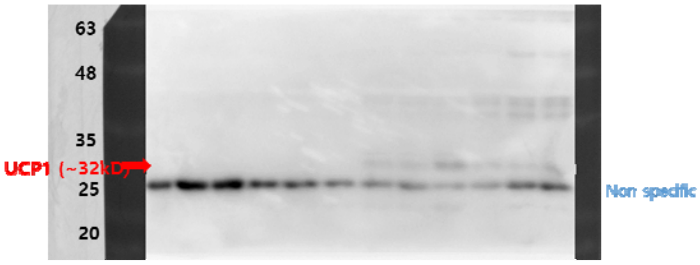

Figure 1E- Tubulin

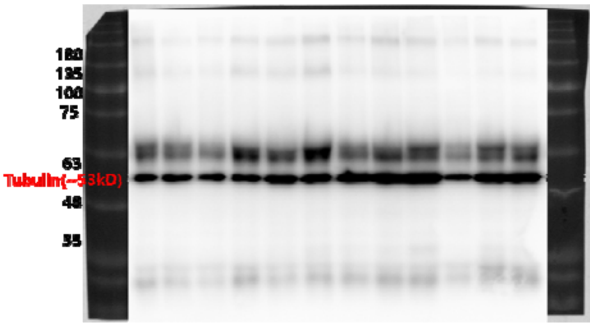

Figure S6. Immunoblots accompanied by size markers (used in Figure 1)

Figure 2D- Cx43

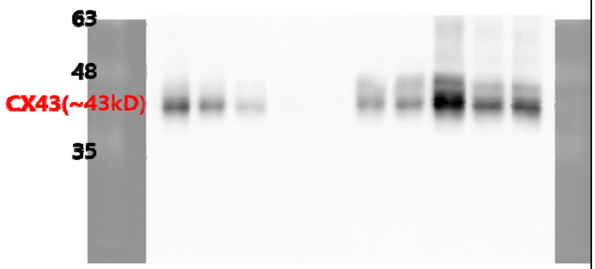

Figure 2D- COX4

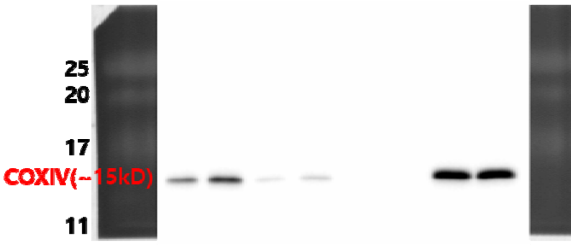

Figure 2D- Na+K+ATPase

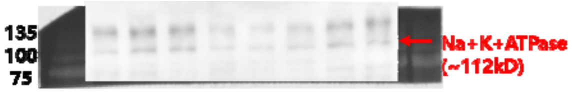

Figure 2F- Cx43

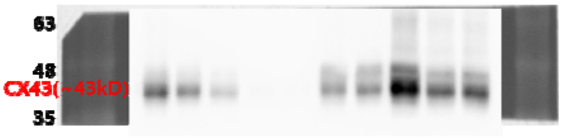

Figure 2F- COXIV

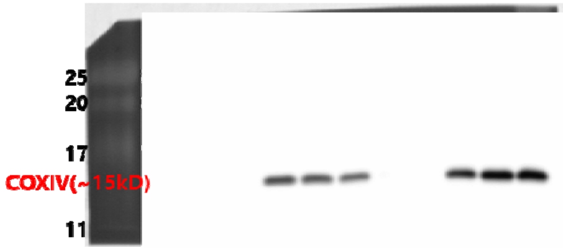

Figure 2F-TOM22

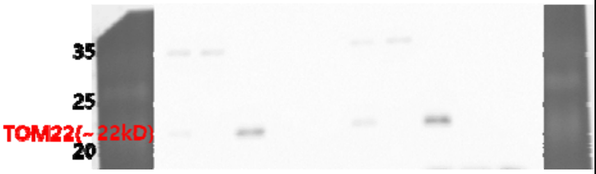

Figure 2F-CAV1

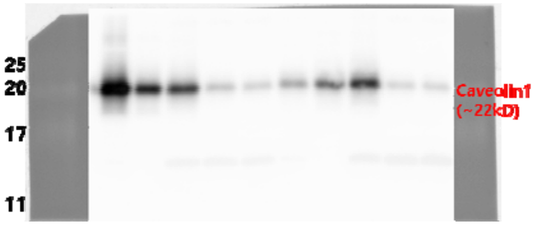

Figure 2F-Na+K+ ATPase

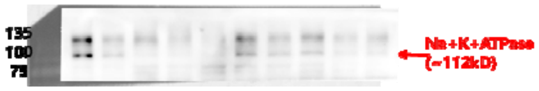

Figure 2F- GAPDH

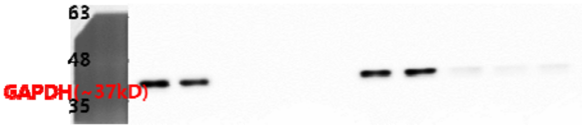

Figure S7. **Immunoblots accompanied by size markers (used in Figure 2)**

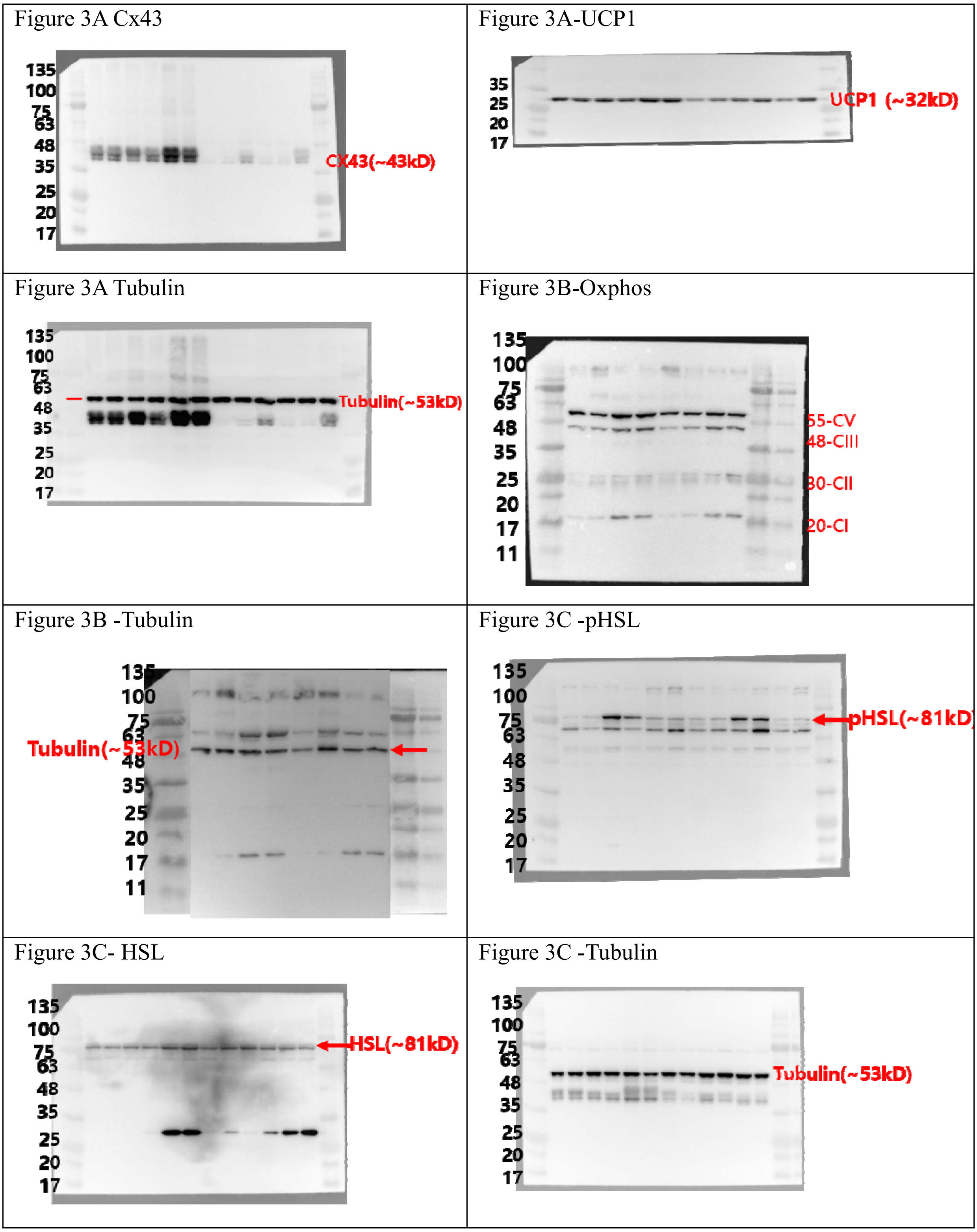

Figure S8. **Immunoblots accompanied by size markers (used in Figure 3)**

Figure 4E Cx43	Figure 4E Tubulin
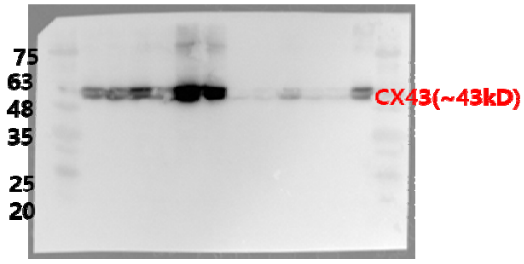	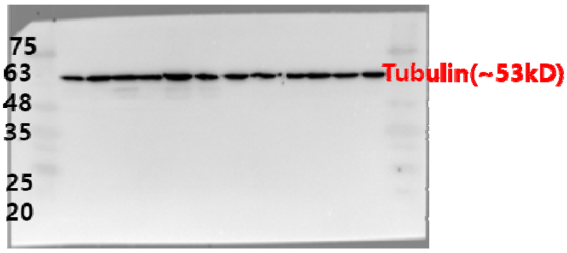
**Figure 4E LC3B**	**Figure 4E LC3B**
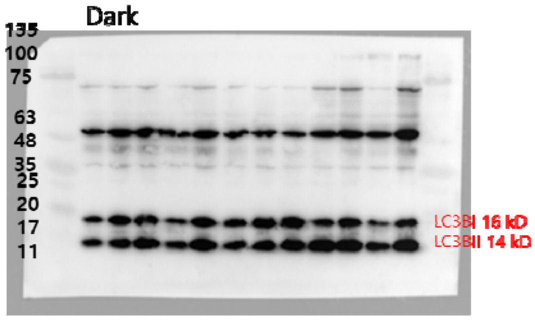	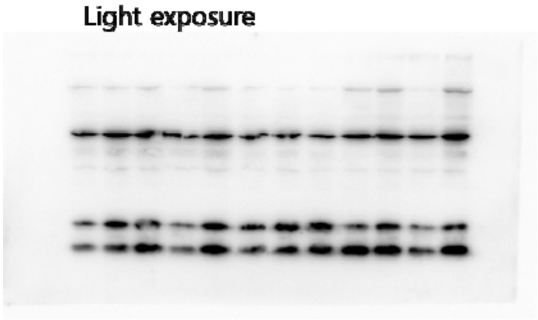

Figure S9. **Immunoblots accompanied by size markers (used in Figure 4)**

Figure 5A –Cx43

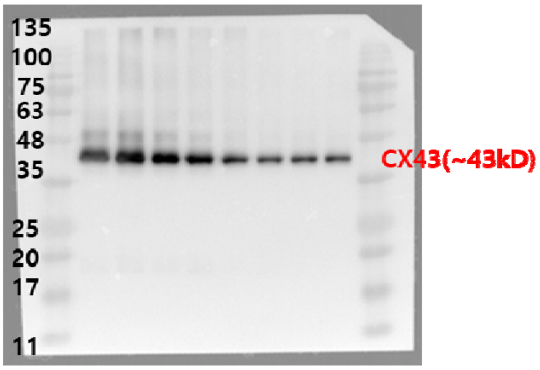

Figure 5A- Oxphose

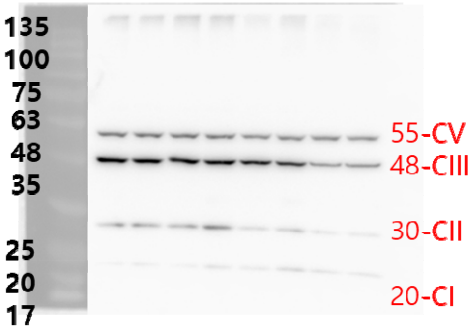

Figure 5A- Tubulin

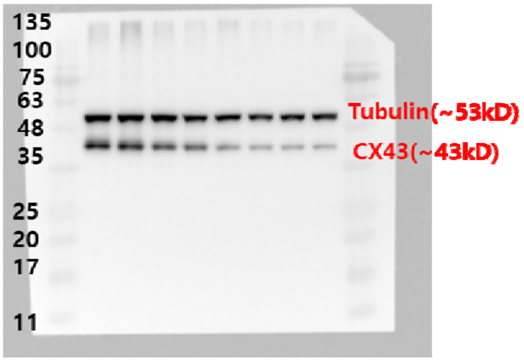

Figure S10. Immunoblots accompanied by size markers (used in Figure 5)

Table S1. List of murine primers used for real time qPCR

Symbol	Full name	Primer sequence (5'-3')	
		Forward	Reverse
Gjc1	gap junction protein, gamma 1	AAACAACCCCCATGGTCCTC	TTAAATCCAGACGGAGGTCTTCCC
Gjd4	gap junction protein, delta 4	GTCTTGCTCGCATGTACCCT	AGGATCCAAAGCAGGTCAGC
Gja4	gap junction protein alpha 4	GTCAGCCGGGAGATAAAGGC	GCCCATGGGGAGGTAGAAGA
Gjb5	gap junction protein beta 5	CGATGAGTTCTTCCCCGTGT	GCCACATGCATGACCACAAG
Nor1	NADP oxidase regulator-1	GTGTCTCAGTGTCGGGATGG	CGAGGGCTCCTGTTGTAGTG
Plin2	perilipin 2	AGCCAACGTCCGAGATTGTT	ATGCTGCCATTGACCACAGA
Atg9a	autophagy related 9A	GCTATCCCTGTGCTACACCC	GATCTGTGATGCCCCCGTAG
Atg12	autophagy related 12	CCCCAGACCAAGAAGTTGGAA	CCATGCCTGGGATTTGCAGTA
Atg7	autophagy related 7	CTGACCTTCGCGGACCTAAA	GGTCCCCGGATTAGAGGGAT
Atg4b	autophagy related 4B cysteine peptidase	CGGCTGCACTTCCTACTGAT	ACTAGTGGTCTCCAAGCCGA
Naga	N-acetyl galactosaminidase, alpha	GAACTGCATCAGTGAACGGC	CACCAATCCAGCAGTCATCG
Tfe3	transcription factor E3	TCTCAGTTCCCGGGTGTAATC	CAGACGGGAATGGTGCAATC
Ctsz	cathepsin Z	AAACACAACTGCACTGGGAC	CTGAAGGCAAGCACTGTAGAAG
Ctss	cathepsin S	GGATGCTTCATGTGACAAGCTC	TGTGGTGCTCACTCCAAAGA
